# Ist das Gegebene noch zu retten? Über Chancen und Gefahren einer Politisierung der Phänomenologie

**DOI:** 10.1007/s10743-025-09363-5

**Published:** 2025-07-03

**Authors:** Sonja Rinofner-Kreidl

**Affiliations:** Department of Philosophy, University of Graz, Heinrichstraße 26/V, 8010 Graz, Austria

**Keywords:** Interne vs. externe Politisierung, Zickzack-Methode, Multiperspektivität, Standpunktpluralismus, Tiefe Sozialität, Epistemische vs. soziale Autorität, Selbstwiderlegung radikaler externer Vernunftkritik

## Abstract

Dieser Essay zielt darauf, eine Mehrdeutigkeit der Rede von „Politisierung“ herauszuarbeiten, die sowohl für die Selbstkritik phänomenologischen Denkens als auch für die Kritik der Phänomenologie als eines öffentlichen Diskurses von Bedeutung ist. Die fraglichen Unterschiede betreffen das Verständnis dessen, was infolge einer Politisierung des Denkens anders konzipiert, kontextualisiert oder interpretiert wird, ob eine Politisierung intrinsisch oder extrinsisch motiviert und begründet ist und wie Art und Reichweite der daran geknüpften Ansprüche argumentiert werden können. Zwei Optionen werden erörtert: (1) eine interne Politisierung, die als phänomenologisch-methodischer Umgang mit Gegebenem und als kollektiv praktizierter Evidenzstil charakterisiert wird; (2) eine externe Politisierung, die sich als standort- und kontextabhängige weltanschauliche Idee und Praxis darstellt. Letztere versteht sich als eine politische Forderung und Erwartung, die eine thematisch einseitige bzw. verengte und / oder unreflektierte, unkritische, womöglich vorurteils- und ressentimentgeleitete phänomenologische Untersuchung korrigiert. Es wird argumentiert, dass interne Politisierung auf einer Metaebene stattfindet, auf der über Natur und Selbstbegrenzung der phänomenologischen Analyse nachgedacht wird. In konkreten Phänomenanalysen schlägt sich interne Politisierung lediglich indirekt, über deren methodische und theoretische Rahmung, nieder. Interne Politisierung ist mit autonomer Vernunftausübung verträglich. Dies gilt nicht für jede Form externer Politisierung, die als direkter Eingriff auf der gegenständlichen Ebene, im Zuge der Interpretation konkreter Phänomene, erfolgt. Zu klären ist: Wie können zulässige und eventuell unabdingbare Formen von Politisierung von unzulässigen unterschieden werden? Unter welchen Bedingungen unterliegt die Politisierung des Denkens einem Selbstwiderlegungseinwand?

## Motivation und Kontextualisierung des Problems

Politisierung ist ein dominanter Zug gegenwärtiger Geisteshaltung und Mentalität. Abgesehen ist es dabei auf Konkretisierung, Differenzierung und Engagement. Unterliegt ein philosophisches Denken diesem Anspruch, so wird erwartet, dass es—umfassender, deutlicher, in höherem Maße—auf die Anforderungen seiner Zeit und Kultur reagiere. Aktuell koinzidiert diese Erwartung mit der Idee, dass die Qualität und Legitimität philosophischer Problemanalysen nicht losgelöst von der Herkunft, sozialen Position und kulturellen Identität derer betrachtet werden sollte, welche die betreffenden Analysen durchführen.^1^ Was genau die Anforderung besagt, man müsse (s)ein Denken politisieren, ist nicht ohne Weiteres klar. Ein nahe liegender Vorschlag lautet: Politisierung des Denkens zielt auf dessen Einbettung in einen öffentlichen politischen Diskurs, der gemäß der Idee demokratischer Meinungsbildung eine Vielfalt von Standpunkten und Interessen abbildet. Entsprechend richtet sich das Augenmerk stärker auf die handlungspragmatischen Konsequenzen eines Denkens als auf die immanenten Begründungsansprüche, mit welchen sich dasselbe in wechselnden theoretischen Zusammenhängen konfrontiert sieht. Wichtiger als die Verortung in Theoriezusammenhängen ist, so der Vorschlag, die Verbreitung und Aneignung eines Denkens in konkreten historisch-kulturellen Kontexten. Es sind auch stärkere Lesarten der Idee eines politisierten Denkens denkbar. So könnte argumentiert werden, dass eine—richtig verstandene und wirksame—Politisierung erst dann vorläge, wenn die betreffenden historisch-kulturellen Kontextualisierungen Rückwirkungen auf scheinbar rein immanente Theoriebildungen und Theorien zeitigten. Diese Lesart ist stärker, da es sich bei der fraglichen Politisierung nicht bloß um eine Interessenverschiebung mit Bezug auf zwei Bereiche handelt, die in ihrer Eigenart und Verschiedenheit unangetastet bleiben. Es handelt sich vielmehr um eine Neubestimmung ihres Verhältnisses: um Unter- oder Überordnung. Die zweite, stärkere Lesart der Forderung nach Politisierung des Denkens wirft ein helleres Licht auf die Tragweite der Fragestellung. Diese Fragestellung sollte auch in die Selbstreflexion philosophischer Theoriebildung Eingang finden.

In den ersten Dekaden des 20. Jahrhunderts, der Gründerzeit der modernen Phänomenologie, charakterisiert Husserl die Selbstreflexion philosophischer Theoriebildung als einen dynamischen Prozess, der in einem Hin und Her zwischen Methodenreflexion, konzeptioneller Arbeit und konkreten Analysen verläuft. Das Hin und Her zielt darauf, originäre und evidente Erfahrung von Gegenständlichem aller Art zu erlangen. Die folgenden Überlegungen zeichnen in einer weiterführenden Interpretation das nach, was Husserl eine „Zickzack“-Methode nennt, und was eine Form der geistigen Bewegung, aber auch ein Analyseverfahren darstellt. In der *Krisis*-Schrift wendet er diese Methode auf die verstehende Erschließung des Sinnes der modernen Wissenschaft aus der hermeneutischen Spannung zwischen historischem Ursprung und gegenwärtiger Entwicklungsgestalt an.^2^ In anderen Kontexten erschließt sich das Verhältnis von Erfahrung und Reflexion im Zickzack, indem sich eine horizontale, zeitliche Dimension mit einer vertikalen, systematischen verknüpft. Indem die Analyse fortschreitet, im Hin und Her zwischen Erfahrung und Reflexion, differenziert sich der Zugriff auf das Untersuchte und gewinnt der Umgang mit ihm zugleich eine Tiefendimension, die er im Erstkontakt nicht hatte und nicht haben konnte. So führt die Exploration des Gegenstandes implizit ihre „Historie“ mit sich. „Aber es liegt überhaupt in der Natur der Phänomenologie, daß sie schichtenweise von der Oberfläche in die Tiefe dringt. […] Produkte einer ersten Analyse bedürfen einer neuen reinigenden Destillation, die neuen Produkte wieder, bis das letzte völlig rein und klar gewonnen ist.“ (Husserl, 1973b, 12) Dabei entspricht der Differenzierung und Vertiefung, die auf Seiten des Gegenstandes gewonnen wird, eine kritische Einstellung auf Seiten des erfahrenden Subjekts. Dieses vollzieht „ein Urteilen sozusagen *im Zickzack*, ein zunächst geradehin auf Selbstgebung lossteuerndes, aber dann in der Weise der Kritik auf die schon gewonnenen vorläufigen Ergebnisse zurückgehendes, wobei aber die Kritik selbst wieder in Kritik genommen werden muß, und aus gleichen Gründen. Den Wissenschaftler leitet also die Idee einer auf dem Wege der Kritik erreichbaren vollkommenen oder in systematischen Stufen zu vervollkommnenden Evidenz mit dem Korrelat eines erzielbaren oder approximierbaren wahrhaften Seins […].“ (Husserl, 1974, 130).

Der Ruf nach Politisierung reagiert auf einen Einwand gegen phänomenologisch verfahrende Sinnanalysen: dass diese nicht nur lebensfern und „zahnlos“ seien, sondern darüber hinaus unter dem Verdacht des Dogmatismus und Autoritarismus (z. B. in Gestalt von Präsentismus, Eurozentrismus und Kolonialismus) stünden,^3^ sofern nicht explizit gemacht und kritisch betrachtet würde, von welchen konkreten Subjekten mit welchen sozial, kulturell, ethnisch und genderbezogen diversifizierten Erkenntnis- und Handlungsinteressen ein Phänomen untersucht würde. Denn es ist, so die Überlegung, eine fragwürdige Abstraktion bzw. eine Illusion, von einem anonymen Erkenntnissubjekt zu sprechen und dieses im Hinblick auf seine intellektuellen Leistungen und verfügbaren Evidenzen idealisierenden Annahmen zu unterwerfen.^4^ Eine realistische Charakterisierung von Erkenntnis- und Handlungssubjekten erfordert umfängliches Wissen über die faktische Konstitution und Vielfalt menschlicher Lebewesen und Lebensformen. Sie erfolgt auf Basis kulturanthropologischer, soziologischer, historischer, psychologischer und anderer einzelwissenschaftlicher Studien. Der Rückbau idealisierender Annahmen mit Bezug auf Erkenntnis- und Handlungssubjekte und die Annäherung an reale Menschen, deren Fähigkeiten und Defizite, hat die Philosophie im auslaufenden 20. und beginnenden 21. Jahrhundert unter verschiedenen thematischen Gesichtspunkten (z. B. bezüglich der Abgrenzung von Rationalität und Irrationalität,^5^ Normalität und Anormalität^6^) beschäftigt. Das anhaltende Interesse an der Konkretion der Subjekte und der Erkenntnis- bzw. Handlungskontexte speist sich gegenwärtig u. a. aus wissenschafts- und ideengeschichtlichen Untersuchungen, aus dekonstruktivistischen Philosophien und Tugendethiken, aus Beiträgen der *Critical Phenomenology* und aus Responsivitäts- und Alteritätstheorien.^7^ Überdies hat sich mittlerweile eine Phänomenologie des Politischen bzw. eine politische Phänomenologie etabliert, welche die Pluralisierung der Subjekte, Statusfragen, Machtverhältnisse und Chancenungleichheit ins Zentrum rückt, auch im Hinblick auf die Entwicklung und Betätigung jener intellektuellen und sozialen Fähigkeiten, die für die demokratische Gestaltung der Gesellschaft und den öffentlichen Diskurs unverzichtbar sind.^8^

In jüngerer Zeit geht es in den anhängenden Debatten nicht nur darum, Vielfalt und Diversifikation zur Kenntnis zu nehmen. Es geht vor allem darum, die Verzerrungseffekte, die auf eine diesbezügliche *Nicht*kenntnisnahme folgen, genauer zu untersuchen und insbesondere auf rassistische, antifeministische, politisch-reaktionäre oder religiös-extremistische Vorurteile und Ressentiments zurückzuführen. Diese Problemlagen finden ihr Echo in der sozialen und politischen Erkenntnistheorie, die mit Fake News, Verschwörungstheorien, dem schwindenden Vertrauen in die Urteilsbildung anderer (z. B. von Expertinnen) oder in das Wahrheitsziel überhaupt, ebenso befasst ist wie mit epistemischen Lastern, schuldhaftem Nichtwissen, biasbedingten Irrtümern und Blindheiten.^9^ Dass derartige Untersuchungen boomen, ist keine Überraschung—„in the current crisis over the relevance of truth and knowledge in contemporary politics“ (Edenberg & Hannon 2021, 2), die sich in einer „intensified epistemic anarchy“ (ibid.) bekundet.

Das obige Bild bleibt skizzenhaft. Dennoch verdeutlicht es, wie sich das Interesse an der Historisierung, Kulturalisierung und Sozialisierung von Erkenntnis- und Handlungssubjekten in einer *polyphonen* („vielstimmigen“) Rede vom Subjekt äußert. Um das weite Spektrum einer Revision der vormals *uniformen* neuzeitlich-aufklärerischen Rede vom Erkenntnissubjekt zu adressieren, spreche ich im vorliegenden Zusammenhang von einer „Politisierung“ der Phänomenologie. Die Phänomenologie politisiert sich in dem Maße, in dem sie bereit ist, die oben angesprochene Diversifizierung der Subjekte, welche phänomenologische Studien betreiben und/oder in den betreffenden Analysen als Untersuchungsgegenstände vorkommen, in ihre Forschungsagenda aufzunehmen.

Die vorliegende Auseinandersetzung unterliegt gravierenden Einschränkungen. Ich werde mich mit den Subjekten der Phänomenologie nur insofern befassen, als sich diese in der aktiven Rolle der Untersuchungssubjekte—nicht in der passiven von zu untersuchenden Gegenständen—finden. „Aktive Rolle“ des Subjekts meint nicht, dass jederzeit ein Subjekt oder verschiedene Differenzierungen von Subjektfunktionen (z. B. Husserls Unterscheidung von fungierendem und thematischem Ich oder die *Krisis*-Debatte über die sogenannte *Paradoxie der Subjektivität*) bzw. das Selbstverhältnis und die Selbstbeschreibungen von Subjekten unter methodischen Gesichtspunkten Gegenstand der Betrachtung sind. Subjektaktivitäten in dem hier gemeinten Sinn liegen implizit auch dann vor, wenn scheinbar rein sachbezogene zentrale Konzepte der phänomenologischen Methode wie Anschauung, Evidenz, Beschreibung und Reduktion behandelt werden. Im Rahmen einer phänomenologischen Subjektivitätsforschung gilt das vordringliche Interesse dem Gegenstandsbezug der Methode bzw. ihrer Angemessenheit mit Blick auf den Gegenstand. Ich werde mich hauptsächlich indirekt auf das aktiv forschende Subjekt beziehen, nämlich insofern sich dessen Verhalten in typischen methodenrelevanten Handlungen, Positionierungen und Dispositionen niederschlägt.

Damit liegt das Augenmerk primär auf methodisch geformten Aspekten der Subjektivität und weniger auf dem, was oft im Zentrum des Interesses steht, wenn die Konkretion und Kontextualität philosophischen Denkens untersucht wird—nämlich die leiblich begründete lebensweltliche Situiertheit des Subjekts. Diese ist, sowohl thematisch-gegenständlich als auch methodisch unbestritten eines der wichtigsten und grundlegendsten Themen der modernen Phänomenologie. Die zugehörige Kernfrage nach den Implikationen der faktischen Konstitution des menschlichen Subjekts für die phänomenologische Methode wurde von Merleau-Ponty formuliert: Wie müssen wir das Subjekt denken bzw. wie muss das Subjekt beschaffen sein, wenn eine Reflexion auf seine eigene Situiertheit wie auch ein konstitutiver Beitrag des Anderen zur Konstitution von Welt und Selbst möglich sein sollen?^10^ Die hier geforderte Reflexion wird näher qualifiziert als eine radikale philosophische Reflexion, welche ihre eigenen Ermöglichungsbedingungen und Grenzen mitbedenken muss. Die folgende Erörterung variiert diese Kernfrage der Selbstaufklärung phänomenologischen Philosophierens im Lichte dessen, was es bedeutet und erfordert, als geistiges Wesen forschend tätig zu sein und aktiv nach Erkenntnis und Wahrheit zu streben. Speziell interessiert folgende Frage: Wie müssen wir die exemplarisch bei Husserl aufgegriffene Methode der Phänomenologie und das phänomenologisierende Subjekt denken, wenn es möglich sein soll, zu einem differenzierten Konzept der Politisierung der Phänomenologie zu gelangen? Es steht mithin nicht die Ambiguität des Subjekts als leiblich-seelisch-geistige Lebenseinheit oder als mundanes *und* transzendentales Subjekt im Fokus, sondern die Kontextgebundenheit der menschlichen Vernunft, sofern es um Ausdruck, Artikulation und Realisierung im Vernunftgebrauch, als Ausübung der Vernünftigkeit einzelner Subjekte, geht. Mit Bezug darauf werde ich die These vertreten, dass Autonomie des Denkens und Politisierung des Denkens einander nicht prinzipiell ausschließen, sofern ein entsprechend differenziertes Konzept der Politisierung zugrunde gelegt wird.

Mit dem Fokus auf das phänomenologisierende Subjekt, *soweit es methodisch involviert ist*, fällt ein weites Untersuchungsfeld der politischen Phänomenologie fort, die das oben skizzierte Problem der Diversifikation im Feld des sozialen und politischen Handelns, unter anderem mit Blick auf Fragen der Normierung und Gestaltung verschiedener Gesellschaftsordnungen, bearbeitet. Die indirekte, methodenvermittelte Referenz auf das phänomenologisierende Subjekt interessiert sich vornehmlich für die Explikation dessen, was in konkreten Phänomenanalysen als *tacit knowledge einer Methodenpraxis der Phänomenologie* fungiert. Diesbezüglich zentrale Konzepte sind unter anderem: Standpunkt, Einstellung, Perspektive und Horizont.^11^ Mit diesem Fokus spreche ich im Folgenden über die Politisierung der Phänomenologie.

Der Argumentationsgang stellt sich wie folgt dar. Ausgehend von der obigen Problemexposition präzisiert der zweite Abschnitt die vorliegende Verwendung des Terminus „Politisierung“, indem eine enge von einer weiten Bedeutung unterschieden und die Frage der Politisierung einerseits auf gegenständlicher und andererseits auf einer Metaebene situiert wird. Unter Voraussetzung dieser Differenzierungen erörtert der dritte Abschnitt Chancen und Gefahren einer Politisierung der Phänomenologie. Im vierten Abschnitt wird die zentrale These entwickelt, dass Autonomie der Vernunft mit interner Politisierung (im vorliegenden Verständnis) nicht nur kompatibel ist, letztere vielmehr unverzichtbar ist, da sie Bestandteil eines autonomen Vernunftgebrauchs ist. Dagegen ist radikale externe Politisierung mit Idee und Gebrauch autonomer Vernunft unverträglich. Vor diesem Hintergrund nimmt der fünfte Abschnitt den roten Faden der Auseinandersetzung im dritten Abschnitt wieder auf, um darüber zu reflektieren, wie Gefahren der Politisierung vermieden und Chancen genützt werden können und wie Phänomenologinnen in Zukunft auf die Dringlichkeit des Politisierungsthemas reagieren könnten.

Wenden wir uns vorerst dem Konzept der Politisierung zu und versuchen wir, dieses nach einigen Aspekten differenzierter zu fassen.

## Politisierung: eng vs. weit, intern vs. extern, auf gegenständlicher vs. Metaebene

Ich verwende den Terminus „Politisierung“ in einem engen und in einem weiteren Sinn. Im engen Sinn schließt die Rede von „Politisierung“ an das herkömmliche, alltägliche Verständnis von Politik und, im theoretisch-philosophischen Kontext, an die gegenwärtige Debatte um die Diversifikation epistemischer und praktischer Akteure und die faire Berücksichtigung aller anhängenden sozialen, kulturellen, ethnischen, genderbezüglichen und anderer Differenzen und Interessen an. In einem *vom Standpunkt dieser Debatte aus gesehen* weiteren Sinn spreche ich von „Politisierung“ mit Bezug auf die phänomenologische Methode, sofern diese implizit oder explizit Gebrauchsweisen von Methodenkonzepten legitimiert und Interpretationsspielräume eröffnet, die eine Sozietät von Erkenntnissubjekten in Anspruch nehmen, welche potentiell in Evidenz- und Einsichtskonflikte geraten und Modi finden müssen, sich über diese zu verständigen und sie soweit als möglich beizulegen. Eine „Politisierung“ erfolgt hier über die methodischen und theoretischen Ansprüche, denen sich eine Erkenntnisgemeinschaft einerseits ausgesetzt sieht, welche sie jedoch andererseits selbst instituiert und über welche sie auch Identitätsbildung betreibt. In diesem philosophischen und methodenbezogenen Sinn verwende ich „Politisierung“ mit Blick darauf, dass eine Pluralisierung von Erkenntnisinteressen, Interpretationen und methodenausübenden Subjekten in Anspruch genommen und eventuell explizit anerkannt wird. So verstanden ist die Phänomenologie immer schon „politisiert“. Sie generiert kontext- und theorierahmenabhängige Problemformulierungen und reflektiert über das Zusammenspiel deskriptiver und normativer Komponenten in der Analyse verschiedener Arten und Formen von Intentionalität; sie inszeniert und kontrolliert Einstellungswechsel, welche wiederum einen Wechsel der Erscheinungsweise der zu untersuchenden Gegenständlichkeiten bedingen; sie engagiert sich in arbeitsteiligen Explorationen verschiedener Gegenstandsfelder und Phänomene; sie praktiziert eidetische Variation und untersucht Zug um Zug damit das methodische Zusammenspiel von Anschauung, Einstellung und Beschreibung; sie kultiviert eine Sprache der deskriptiv-phänomenologischen Analyse. Ich spreche von „interner Politisierung“ im Sinne der Anforderung, die Komplexität gegenständlicher Erscheinungen nach allen relevanten Aspekten zu untersuchen und in einer Gemeinschaft von Forscherinnen Erfahrungsprozesse und -analysen auf Basis eines geteilten Verständnisses für methodische Regularien und normative Hintergrundüberzeugungen zu organisieren, durchzuführen und philosophisch auszuwerten.

Entscheidend mit Bezug auf das hier vorgeschlagene Verständnis einer internen Politisierung der Phänomenologie ist, dass sich die methodisch geleiteten Bewährungsprozesse, welche sich auf Anschauungen und Beschreibungen einer Vielzahl von Phänomenen beziehen, der Normierung durch die betreffende Sache selbst unterstellen.^12^ Die Anerkennung dessen, dass es die zu erforschende Sache selbst ist, welche das Maß (sc. die Art und Weise) ihrer Erkennbarkeit vorgibt, erfolgt konsensual.^13^ Diese normative Vorgabe erhält aufgrund des fortgeschriebenen Konsenses der Phänomenologinnen den Status einer Normalität. Normalität äußert sich demnach in permanenten Normalisierungsprozessen, die das, was eine an der Sache zu bewährende Einsicht und Überzeugung ist, in eine Selbstverständlichkeit verwandeln.^14^ Diese bleibt jedoch befragbar. Sie kann jederzeit auf ihre sachliche Grundlage hin überschritten werden und muss im Falle widersprechender oder unklarer Evidenzlage auf diese hin überschritten werden.^15^ Die normative Vorgabe, über das jeweilige gegenständliche Erkenntnisinteresse hinaus gedacht, gründet in der Anerkennung eines Erkenntnis- und Wahrheitsziels überhaupt.^16^ Was liegt darin? Warum handelt es sich hier um den—wie ich vorhin sagte—entscheidenden Umstand?

Es geht hier um das, was die Erkenntnisgemeinschaft der Phänomenologinnen primär eint. Mit Bezug auf die Realisierung des Erkenntnis- und Wahrheitsstrebens ist das eine Überzeugung und Haltung, die darin gründet, eine spezifische Einheit/Vielheit-Struktur als Grundbedingung des Bezugs auf Gegenständlichkeit wie auch jeder möglichen Selbstbezüglichkeit des „Habens“ von Welt anzuerkennen. Diese Überzeugung und Haltung realisiert, dass ein und dasselbe x *als Etwas*, d. h. als ein relativ stabiles, über den Zeitlauf hinweg adressierbares Etwas, gerade deshalb erfahrbar ist, weil es in verschiedener Weise, in verschiedenen Ansichten, aus verschiedenen Perspektiven, in verschiedenen Aufeinanderfolgen von Erscheinungsweisen adressiert (wahrnehmend erfasst, beurteilt, erinnert etc.) werden kann. Denn Gegenständlichkeit impliziert Erlebnistranszendenz: die Nichtreduzierbarkeit des Gegenstandes auf ein momentanes Wahrnehmungserlebnis. *Erfahrbar* ist die so verstandene gegenständliche Transzendenz nur dann, wenn das betreffende Etwas im Zeitablauf *eines* Bewusstseins bzw. in der gleichzeitigen Erfahrung durch *mehrere* Bewusstseinsträger als dasselbe *wiedererkennbar* ist—nämlich: in seinem dem jeweiligen Wahrnehmungserlebnis gegenüber selbständigen Soseinsanspruch. Im individuellen und kollektiven Erfahrungsprozess erhält diese Selbständigkeit des „Gegen-Standes“ Gewicht aufgrund der Explorier- und Bewährbarkeit seiner sachhaltigen Bestimmungen, das heißt aufgrund der ihm jeweils eigentümlichen gegenständlichen Komplexität. Was die Pluralität der mannigfachen Erscheinungsweisen des Gegenstandes auf Seiten der erfassenden Subjekte gewährleistet, ist eine Varianz von Einstellungen, Interessen und Standorten—sowohl wörtlich verstanden, als auch im übertragenen, z. B. imaginären Sinn. (Während ich auf den blühenden Kirschbaum in meinem Garten blicke, stelle ich mir vor, wie sich dieser für meinen Nachbarn darstellt, der mir gerade von drüben, vor seinem Haus stehend, zuwinkt.) Was im Hervorbringen einer Erscheinungsvielfalt, unangesehen der Irrtumsanfälligkeit jeder gegenständlichen Bezugnahme, *der Möglichkeit nach* Objektivität sicherstellt, ist das, was ich vorhin die „normative Vorgabe“ und deren nachkommende, auf intersubjektiven Konsens *hinarbeitende* Bestätigung genannt habe. Das heißt: Phänomenologinnen teilen—in fortlaufender Forschungspraxis häufig im Sinne eines *tacit knowledge*—die Einsicht, dass es nicht der jeweilige Standort des anschauenden und beschreibenden Subjekts ist, der das letzte Wort mit Bezug auf gegenstandsbezogene Geltungsansprüche hat, sondern stets der kognitive Gehalt bzw. der in wechselnden Erscheinungsweisen sich bekundende Gegenstand—natürlich der Gegenstand gemäß der Art und Weise, wie er sich jeweils (unter der Bedingung identisch festgehaltener gegenständlicher Referenz) zeigt bzw. gibt. Letzteres bedingt im Rahmen der *Relationalität des Erscheinens von etwas für jemand* die Einbeziehung des Standortes der erfassenden Subjekte.^17^ Dennoch gilt als normierend stets das, *was* von diesem Standort aus so-und-so erscheint und *was* in der jeweiligen Erscheinungsweise als etwas aufgefasst ist, das ebenso von anderen Standorten aus erscheinen könnte und prinzipiell von *irgendeinem* Standort aus „erscheinbar“ sein muss.

Die vorweg anerkannte „Normierungsmacht“ des Gegenstandes verhindert, dass die transzendentale Subjektivität, das heißt die intentionale Relationalität der Gegebenheitsweise des Gegenstandes, in einen Subjektivismus im Sinne der Prädominanz des jeweiligen subjektiven Standortes kippt.^18^ Multiperspektivität und eine über multiperspektivische Gegebenheit(sweis)en angenäherte Universalität liegen in der Struktur des Erfahrungsprozesses. Dies zeigt sich jedoch nur dann, wenn als dominanter Faktor das in Erfahrung zu Bringende, der Gegenstand, und nicht der zufällige Standpunkt des jeweiligen Erfahrungssubjekts anerkannt wird. Eine Wechselverständigung, die eine Pluralität von Subjekten und eine Pluralität von Standpunkten involviert, kann sich nur dann als eine Form der Erfahrung etablieren, wenn gemeinsame („geteilte“) gegenständliche Referenz möglich ist und im Sinne einer *prima facie* Evidenz unterstellt wird. Das schließt nicht aus, dass diese Unterstellung im Einzelfall aus nachvollziehbaren Gründen scheitert.

Zu den methodischen Regularien, welche eine systematische Erkundung gegenständlicher Erscheinungen ermöglichen, gehören Variationen von Erscheinungsweisen, die mit Hilfe von (thematischen) Einstellungsänderungen gezielt herbeigeführt werden. Abgesehen von den gegenstandsbezogenen Effekten von Einstellungsänderungen als explorativer Methode ist Husserls Einstellungslehre auch im Hinblick auf das epistemische und praktische Selbstverhältnis der phänomenologisierenden Subjekte folgenreich. Von Zeit zu Zeit erneuern wir unser Erkenntnisinteresse, indem wir *ad fontes* gehen und die Frage stellen: Wie kommt das Subjekt ins Spiel, wenn Gegenstände untersucht werden? Wie hängt das eine mit dem anderen zusammen? Erst vor diesem Hintergrund klärt sich die Bedeutung der Einstellung im Kontext gegenständlicher Explorationen. Gemäß der Konzeption der Intentionalität fungiert die gegenständliche Ausrichtung in verschiedenen Typen von Bewusstseinserlebnissen „selbstvergessen“. Entsprechend sind intentionale Erlebnisse transparent. Machen wir uns das anhand eines Beispiels klar. In üblichen Wahrnehmungssituationen ist das Subjekt ausschließlich mit dem so-und-so-erscheinenden Gegenstand befasst; es ist weder mit sich selbst als Wahrnehmendes noch mit medial fungierenden Sinngehalten befasst, welche in das Wahrnehmungsgeschehen hineinverflochten sind. So sehe ich z. B. den rot gefärbten Stuhl und nicht Roteindrücke oder Rot-Sinnesdaten; ich sehe den Stuhl und nicht meine Stuhlwahrnehmung bzw. bestimmte Aspekte derselben.^19^ Es kann natürlich der Fall sein, dass ich aufgrund bestimmter Umstände (z. B. schlechter Lichtverhältnisse) daran zu zweifeln beginne, ob der Stuhl, den ich vor mir sehe, tatsächlich rot und nicht etwa blaßrosa oder pfirsichfarben ist. In diesem Fall fokussiere ich auf einen bestimmten Aspekt, die Färbung, indem ich meine thematische Einstellung auf den Gegenstand ändere. Ich versuche, meine Zweifel zu beseitigen, indem ich systematisch Wahrnehmungsbedingungen—und folglich Erscheinungsweisen—variiere (z. B. den Stuhl aus dem abgedunkelten Raum ins Freie trage), um meine vormaligen Farbeindrücke zu verifizieren oder zu korrigieren.

Was ist nun der für das Selbstverhältnis relevante Aspekt von Husserls Einstellungslehre?^20^ Das Faktum, dass das Subjekt in bestimmter Weise eingestellt ist, kann ihm selbst nur anhand von Einstellungsänderungen im Verlauf seiner Erfahrung konkreter Gegebenheiten von etwas aufgehen, indem es die Korrelation zwischen Einstellungsänderungen und Veränderungen der Erscheinungsweise (bei ansonsten relativ stabilen Bedingungen des gegenständlichen Bezugs) entdeckt. Dieser Sachverhalt kann generalisiert werden. Ohne primären Weltbezug hätte die Ausbildung von Selbstbewusstsein für menschliche Subjekte keine erfahrungsmäßigen Anhaltspunkte. Ohne den Anker einer—mit anderen teilbaren—gegenständlichen Referenz könnten wir kein differenziertes und epistemisch belastbares Verhältnis zu uns selbst als leiblich-seelisch-geistigen Wesen entwickeln. Intentionalität ist eine Ermöglichungsbedingung dafür, ein politisches Wesen im weiteren Sinn zu werden—sowohl im Hinblick darauf, dass unser Sprechen, Wollen und Handeln einen Inhalt braucht, um den es sich im sozialen Wettbewerb des Denkens und Argumentierens zu streiten lohnt: was er bedeutet, ob er realisierenswert ist u. dgl.^21^; als auch im Hinblick darauf, dass wir, um als politische Wesen agieren zu können, imstande sein müssen, ein differenziertes und kritisches Selbstverhältnis auf Basis der Erfahrung einer Pluralisierung von Standpunkten zu entwickeln. Pluralisierung von Standpunkten umfasst hier zweierlei: meine Erfahrung, dass ich, erstens, zu einem beliebigen Gegenstand x diachron verschiedene Standpunkte einnehmen kann wie auch, zweitens, dass andere in zeitgleich stattfindenden Erfahrungen andere Standpunkte zu x einnehmen wie ich selbst. Meiner selbst als Erfahrungssubjekt über die Pluralisierung von Standpunkten bewusst zu werden, schließt ein, dass ich mir selbst quasi als eine stets andere begegne, die mit gegebenen Gegenständen fortlaufend andere, neue Erfahrungen macht, dass ich also in gewisser Weise eine andere werde, indem ich doch ich selbst bleibe; wie auch, dass es andere gibt, die dem Gehalt nach andere Erfahrungen machen, welche aber den meinen material und strukturell hinreichend ähnlich sind, so dass wir uns nicht nur darüber verständigen können, auf dasselbe x bezogen zu sein, sondern auch darüber, *als was* wir x verstehen. („Können“ bezeichnet eine Möglichkeit, nicht etwas, das jederzeit und hinderungsfrei tatsächlich vorliegt.)

Die Pluralisierung der Standpunkte, sowohl gemäß der jeweils eigenen Erfahrung als auch in Erfahrungsgemeinschaften mit anderen, ist eine basale Ermöglichung von Welthabe im Lichte der Idee der Bewährbarkeit gegenständlicher Intentionen, im Lichte der Idee von Objektivität. Zugleich liegt darin die Entdeckung der riskanten Lebensform geistiger Wesen wie wir es sind. Denn die interne Pluralisierung der Standpunkte, individuell wie kollektiv, ist auch der Angriffspunkt dafür, dass unsere bisherigen Überzeugungen fragwürdig werden *können*. Die interne Pluralisierung der Standpunkte mit Bezug auf x bietet nichts geringeres als die erfahrungsvermittelte Einsicht in die Fallibilität der eigenen Überzeugungen. Auch wenn ein Wechsel des Standpunkts gegenüber x die vormalige Überzeugung mit Bezug auf x *nicht notwendigerweise* in Frage stellt, so eröffnet die neue Sichtweise auf den gemeinten Gegenstand doch die Möglichkeit der Nichtübereinstimmung mit bisherigen Erfahrungen. Das Risiko betrifft jedoch nicht nur gegenstandsbezogene Irrtümer.

Der Standpunktpluralismus, zu dem uns Form und Inhalt unserer Erfahrung nötigen, bringt auch die Möglichkeit der Selbstdistanzierung mit sich: dass ich mich als die, die eine bestimmte Überzeugung gebildet hat, im weiteren Erfahrungsverlauf quasi „von (halb)außen“—als ich selbst, aber von einem anderen Standort her—betrachten kann. Das kann als eine Selbstdistanzierung und in einem moderaten Sinn als eine Selbstentfremdung charakterisiert werden. Denn ich werde nun auch für mich selbst, analog zum von mir erfahrenen Gegenstand, als eine Einheit / Vielheit-Struktur greifbar. Ich bin dieselbe, aber in graduell verschiedenen Erscheinungsweisen: einerseits mit im Zeitablauf wechselnden Erfahrungsbeständen, sich ändernden Überzeugungen, Wünschen, Werthaltungen (diachron)^22^; andererseits entsprechend der Differenz meines erstpersonalen Bewusstseins (sc. präreflexiven Vollzugserlebens) bzw. meiner reflexiven Selbstwahrnehmung von der Fremdwahrnehmung meines Fühlens, Denkens, Wollens und Tuns durch andere (synchron). Die Einheit / Vielheit-Struktur und der daraus erwachsende Perspektivenwechsel eröffnen epistemisches und moralisches Potential. Freilich *können* sich Erlebnisse von Selbstdistanz und Selbstentfremdung im Kontext individueller Erfahrungsrealität auch negativ, verunsichernd oder gar ängstigend, bemerkbar machen. Dies ist etwa der Fall, wenn sie Glückszustände eines unbefragten Mit-sich-Einsseins aufheben oder sich in ihnen Episoden innerer Inkohärenz zuspitzen. Anders stellen sich Selbstdistanz und Selbstentfremdung, *strukturell und nach der obigen Charakteristik verstanden*, mit Bezug auf die normative Idee des Selbst als einer intellektuellen und moralischen Persönlichkeit dar. Zu verstehen, dass ich gemäß dieser Idee mehr als nur dieses konkrete Selbst hier und jetzt bin, ist Teil meiner Integrität als eines geistigen und moralischen Wesens. Als solches agiere ich als ein *erweitertes Selbst*.^23^ Was heißt das und inwiefern ist das im vorliegenden Sinn strukturell ermöglicht?

Selbstdistanzierung und Selbstentfremdung sind nicht bloß Ausdruck einer anonym praktizierten Methodologie des Denkens. Insbesondere im Kontext von Ethik und Moralphilosophie werden sie auch als alltagstaugliches Mittel der Objektivierung verstanden. Sie können dazu dienen, Empathie und Perspektivenwechsel einzuüben, sich selbst in der Folge quasi als einen Anderen zu erfahren und sich damit für die Andersheit der anderen zu öffnen. Reife, moralfähige Subjekte sind imstande, in diesem Sinn über sich selbst hinauszugehen.^24^ Als eine tatsächlich erfahrene Weise des Umgangs mit sich selbst sind Selbstdistanzierung und Selbstentfremdung, vor allem in ihrer Wirksamkeit als Selbstkritik, prägende Faktoren der Entwicklung von Personen. Sie sind Teil eines Prozesses, der auch unter dem Gesichtspunkt des Erwerbs intellektueller und ethischer Tugenden beschrieben werden kann. In diesem Sinne jemand Bestimmter zu werden, erfordert, im Dienste einer Sache und in Gemeinschaft mit anderen über sich hinauszugehen.^25^ Dabei wird das Verhältnis zwischen Selbst und anderen im Lichte eines gemeinsamen Bezugs auf die Sache und einer geteilten Zwecksetzung gesehen. Um der zu entdeckenden, zu erkennenden, zu realisierenden Sache willen muss mir die Sicht der anderen wichtig sein und muss ich meine Sicht als eine betrachten, die allen anderen wichtig ist—sofern ich mich überhaupt als ein nach Erkenntnis und Wahrheit strebendes Wesen begreife.^26^ Um an dieser Stelle meine titelgebende Frage aufzugreifen: Das Gegebene zu „retten“, kann nicht die „Heldentat“ einer einzelnen Denkerin sein, die niemals auf sich als ein einzelnes oder vereinzeltes Subjekt reduzierbar ist; es ist ein Gemeinschaftsprojekt, dessen Sinnhaftigkeit im Großen und Ganzen nicht in Frage steht, solange Vernünftig-werden-wollen in kooperativer Erkenntnistätigkeit ein Menschheitsziel ist.^27^ Mit Bezug auf *bestimmte* Gegenstände und Erkenntnisprojekte ist der Ausgang des Prozesses freilich offen.

Um meine vormaligen Überzeugungen in einer nicht strukturell auf Selbstbestätigung angelegten Weise prüfen zu können, muss ich in der Lage sein, eine Semi-Distanz mir selbst gegenüber im oben explizierten Sinn einzunehmen. Insofern geht es bei der Frage der internen Politisierung qua Pluralisierung von Standpunkten generell um die Möglichkeit eines vernünftigen und (im ursprünglichen Wortsinn) kritischen Denkens.^28^ Vernünftiges Denken ist möglich unter der Bedingung einer allseitigen Prüfung und Bewährbarkeit des Gedachten (was immer es ist, das gedacht wird). Gemäß einer phänomenologischen Methodologie im Geiste Husserls ist die Forderung der Bewährbarkeit von der jeweils zu erkennenden Sache—dem gegenständlichen Telos des Erkenntnisstrebens—und letztlich, als formalem Objekt eines jeden solchen Strebens, von der Idee der Wahrheit instituiert. Die Forderung nach Bewährbarkeit stellt eine freiwillige Selbst-Verpflichtung jedes denkenden Subjekts dar, welche zugleich notwendig ist, sofern das Subjekt sich selbst als vernünftig verstehen können soll. Das eigene Denken im Horizont einer offenen Pluralität von Standpunkten zur Disposition zu stellen, verankert das denkende und erfahrende Subjekt in einer *tiefen Sozialität*. „Tief“ bezieht sich darauf, dass die so verstandene Bewährbarkeit als ein in aller (Denk-)Erfahrung wirksames rationales Moment die Anerkennung der Korrigierbarkeit durch Andere einschließt, welche ebenso nach Erkenntnis und Wahrheit streben. Mein Habitus als zum Denken Befähigte, und speziell zu einem in Erfahrung gründendem und erfahrungsgeleitetem Denken, ist konstitutiv und unauflösbar mit dem Bezug auf andere verknüpft. Die Art und Weise, wie die Erfahrung und das Denken *eines* Subjekts im Horizont eines „vielstimmigen“ Bewährungsprozesses exponiert ist, stellt eine fundamentale Bedingung vernünftigen Denkens, Wertens, Wollens und Handelns dar.^29^ Sozialität wird dabei als ein universales Strukturmoment möglicher Erfahrungen in Anspruch genommen. Dieses Strukturmoment ist universal insofern, als es indifferent ist mit Bezug auf bzw. unabhängig von der Mannigfaltigkeit und den Unterschieden konkreter sozialer Lebensformen und sozialer Praktiken, welche sich in verschiedenen historischen und kulturellen Kontexten manifestieren. Selbst wenn ein Subjekt aus zufälligen Gründen isoliert und einsam agiert, ändert dies nichts an seinem impliziten Bezogensein auf andere im Sinne der tiefen Sozialität, sofern es überhaupt Erfahrungen machen kann.

Das Obige entwickelt ein Verständnis von interner Politisierung, indem im Horizont der phänomenologischen Leitidee der Intentionalität über strukturelle Bedingungen von Erfahrung—über die Art und Weise der Involvierung von Subjekten, ihr Selbstverhältnis und ihre tiefe soziale Verankerung—reflektiert wird. Da sich diese Überlegungen nicht direkt auf bestimmtes Gegebenes beziehen, sondern auf die Gegebenheitsweise des Gegebenen, können sie als „Meta-Reflexionen“ charakterisiert werden. Interne Politisierung, soweit sie dem vorliegenden Konzept folgt, wird über Meta-Reflexionen argumentiert. Was meint nun dem gegenüber der Terminus „externe Politisierung“?

In säkularisierten, sozial differenzierten und medientechnisch hoch entwickelten Gesellschaften gibt es keine Deutungsmonopole, und es herrscht Konsens darüber, dass diese nicht wünschbar sind. In Anbetracht der täglichen Informationsflut und im Interesse einer nicht-beliebigen Urteilsbildung ist es umso wichtiger, die verschiedenen Bezugssysteme, in denen Meinungen, Auffassungen und Deutungen generiert werden, als solche auszuweisen und zu unterscheiden. Philosophische Analysen sind im weiteren Sinn Bestandteil dieses gesellschaftlichen Gesamtszenarios, auch wenn sie sich einer separaten, eigenständig entwickelten Theorie und Methodologie verdanken. Soweit sie in diesem größeren Wirkungszusammenhang stehen, können sie nicht bloß als immanente Forschungsangelegenheit betrachtet werden. Dies ist insbesondere dann der Fall, wenn der Untersuchungsgegenstand als gesellschaftsrelevant wahrgenommen wird. Das trifft auf viele kontrovers verhandelte soziale und politische Phänomene zu.^30^ Während wissenschaftliche und philosophische Analysen relativ zum jeweiligen Theoriekontext und aktuellen Stand der Forschung beurteilt werden, verdankt sich die öffentliche Meinungsbildung häufig weniger klar zuordenbaren Bezügen und Deutungshintergründen.

Zeichnen sich die inhaltlichen Bezüge und Motivationen klar ab, stellen sie sich als abhängig von sozialen Positionierungs- und Machtinteressen dar und werden die zugehörigen Interpretationsvorschläge überdies *auf Basis dieser Interessen* als richtig ausgewiesen und immanent-phänomenologischen Analysen korrektiv entgegengestellt, spreche ich von einer (versuchten) externen Politisierung der Phänomenologie. Sofern externe Politisierung lediglich als alternatives Deutungsangebot in Konkurrenz zu philosophischen Analysen auftritt, ist der Anspruch ein begrenzter. Derartige Interventionen können produktiv und erhellend sein, sowohl mit Bezug auf das Verständnis der Sache als auch die dadurch angeregte Selbstkritik. Problematisch ist hingegen, wenn Standpunktpolitik im starken Sinn betrieben und die These vertreten wird, dass (i) jede alltagsweltliche, professionelle, wissenschaftliche oder philosophische Interpretation von (gesellschaftsrelevanten) Phänomenen im engeren Sinn politisch bzw. weltanschaulich konstituiert, motiviert und legitimiert sei bzw. sein müsse, und dass (ii) der so ventilierten Interpretation *aufgrund der hinter ihr stehenden sozialen und politischen Interessen und Machtverhältnisse* Priorität gebühre und sie Richtigkeit für sich beanspruchen könne. Liegt eine derart im starken Sinn außersachlich^31^ und rein politisch bzw. weltanschaulich legitimierte Interpretation vor, welche Anspruch auf Richtigkeit erhebt, spreche ich von „radikaler externer Politisierung“. Diese annulliert der Intention nach jede alternative, und speziell jede wissenschaftliche und philosophische Interpretation, die in der Sache abweicht.

Von externer Politisierung grenzt sich die interne Politisierung der Phänomenologie in markanter Weise ab. Phänomenologisch betrachtet liegt das Politische bzw. die Politisierung (wie sie hier in Erweiterung des üblichen Wortgebrauchs gefasst werden) in dem, als was, in welchen Modi und in welcher Vielfalt wir Erfahrung konzeptualisieren wie auch uns selbst als erfahrende und reflektierende Subjekte konzeptualisieren. Dazu gehört auch das Explizitmachen subjektbezüglicher Voraussetzungen, sofern diese außerphänomenologisch motiviert sind. Das schließt Vorannahmen und Vorurteile verschiedener Art ein wie ebenso politische und weltanschauliche Auffassungen und Identitäten. Generell ist die Thematisierung von verschiedenen Formen der Sinnkonstitution, die menschlicher Weltbeziehung zugrunde liegen, und insbesondere von zuvor „verborgen“ wirksamen Sinnbestandteilen die Zielsetzung der phänomenologischen Reduktion, mit der die Phänomenologie nach Husserls Verständnis die Methodengestalt einer apriorisch-transzendentalen Korrelationsforschung annimmt.^32^ Vor diesem Hintergrund spreche ich über die interne Politisierung der Phänomenologie auf Basis eines bestimmten Erkenntnisstils, bestimmter Methodenideen und Auslegungsspielräume, mit Blick auf eine Sozietät von Forscherinnen, die in bestimmter Weise „trainiert“, „diszipliniert“ und dennoch methodisch auf Erfahrungsoffenheit eingestellt sind.^33^ Es versteht sich, dass der damit verknüpfte Anspruch nicht ist, über politische Phänomenologie als einer speziellen Forschungsdomäne der Phänomenologie zu handeln.^34^ Der Anspruch betrifft vielmehr die Phänomenologie in ihrer Methoden- und Theoriegestalt überhaupt, unangesehen ihrer verschiedenen Forschungsfelder. Interne Politisierung der Phänomenologie tritt nicht als direkte Beeinflussung konkreter gegenständlicher Interpretationen auf. Sie versteht sich als Meta-Reflexion der Implikationen phänomenologischer Methodik (sc. des Insgesamt der gebrauchten Methoden). Dies betrifft speziell die Pluralisierungen und Perspektivierungen mit Bezug auf die involvierten Subjektrollen- und funktionen wie auch die Rückbindung von Anschauung und Beschreibung an die Idee der Phänomenologie, die sich im Gebrauch bestimmter Methoden—der phänomenologischen Reduktion, des thematischen Einstellungswechsels, der eidetischen Variation—niederschlagen.^35^ Interne Politisierung bezieht sich auf die Meta-Reflexion der Subjekt- und Erfahrungskonzepte der Phänomenologie in ihrer Einbettung in eine übergreifende Theorie bzw. eine komplexe Idee philosophischer Forschung. Sie legitimiert sich ausschließlich über diese Anbindung an phänomenologische Selbstaufklärung.

Solange die Phänomenologie ihren selbstgestellten Erkenntnisansprüchen, Methoden und Meta-Reflexionen folgt, ist sie—auch dort, wo sie sich explizit mit politischen Phänomenen bzw. Phänomenen des Politischen in ihrem Gegenstandsfeld auseinandersetzt—apolitisch *im Sinne der oben erläuterten radikalen externen Politisierung*. Sie ist jedoch nicht im herkömmlichen, auf externe Verhältnisse abzielenden Sinn anti-politisch oder unpolitisch. Das entspräche einer resignativen oder indifferenten Flucht in ein Innen- und Privatleben, die politisch leicht zu instrumentalisieren wäre und damit ihrerseits einer (stillschweigenden) politischen Stellungnahme entspräche. Es gibt keinen Ort, an den wir uns zurückziehen könnten, an dem wir nicht immer noch soziale und verantwortliche Wesen und ergo politische Wesen wären. Die obige Argumentation wurde im Rahmen von Husserls Idee der Phänomenologie entwickelt. Mit Blick auf diese lautet die hier vertretene These: Die Phänomenologie ist aufgrund ihres Subjekt- und Erfahrungskonzeptes und unabhängig von ihrem jeweiligen gegenständlichen Fokus intern politisiert. Teil ihres Methodenverständnisses ist, die Differenz von interner und externer Politisierung zu wahren und zu verteidigen.^36^

Zwei Abgrenzungslinien sind mit Blick auf dieses Zwischenergebnis deutlich zu markieren. Beide betreffen die Bedeutung bzw. das Verständnis interner Politisierung. Dass philosophische Selbstreflexion in der oben skizzierten Form einer phänomenologischen Methodenlehre intern politisiert ist, kann auch so erläutert werden, dass das, was hier „politisch“ heißt, allein unter Maßgabe denkerischer Ansprüche ins Spiel kommt, welche auf die intentionale Struktur gegenständlicher Beziehungen und zugehöriger Erfahrungsprozesse respondieren. Methodisch geformte Aspekte der Subjektivität zu untersuchen ist freilich nicht dasselbe wie faktisch vorherrschenden gesellschaftlich-kulturellen Prägungen und sozialen Verhältnissen, in denen konkrete Subjekte existieren, nachzugehen. Dennoch können diese verschiedenartigen Untersuchungsprojekte wechselseitig voneinander lernen und profitieren. Der beiderseitige Vorteil läge darin, über die dichotome Gegenüberstellung einer rein immanenten Theoriebildung und Analyse einerseits und einer rein extern politisch motivierten Kritik andererseits hinauszugelangen. Erst dann kann sich die zugleich grundlegende und zukunftsweisende Frage stellen: Was ist und leistet phänomenologische Forschung jenseits von apriorischem Rationalismus und politischer Instrumentalisierung? Phänomenologie ist Subjektivitätsforschung. Nämlich: eine spezielle Form der Subjektivitätsforschung, welche auf den *Logos* (Idee, Sinn, Gehalt) verschiedener Arten von Gegenständen und verschiedener Formen und Typen von Erfahrung abzielt, soweit sich dieser in intentionalen Strukturen manifestiert. Was das Verhältnis einer so angelegten, methodisch „destillierten“ reinen Bewusstseinsforschung zu wechselnden lebensweltlichen Äußerungen konkreter Intentionalität verständlich macht, ist ein reflexiver Gebrauch der phänomenologischen Methode. Dreh- und Angelpunkt dessen ist die Konzeptualisierung verschiedener Subjektrollen und -funktionen, welche die oben dargelegte Fähigkeit zur Selbstdistanzierung in Anspruch nimmt. Daran hängt die Kontextoffenheit der Phänomenologie, die ihrem rationalen Anspruch nicht entgegensteht, sich diesem vielmehr gerade verdankt. Dass Kontextoffenheit nicht in vollständige soziale „Beherrschung“ und politische Instrumentalisierung des Subjekts kippt, erfordert ein differenziertes Konzept des Selbst. Es erfordert die Idee eines geistigen Subjekts, dessen Vernünftigkeit darin liegt, Vorgegebenes aller Art (Kultur-, Milieu- und Traditionsbedingtes, kollektiv Habitualisiertes, Mainstream-Meinungsbildung etc.) aneignen und aktiv dazu Stellung nehmen zu können.^37^

Die zweite Abgrenzungslinie, die im Auge zu behalten ist, betrifft den Umstand, dass der Fokus auf methodisch geformte Aspekte der Subjektivität eine gewisse Entindividualisierung zur Folge hat.^38^ Diese Entindividualisierung betrifft die Ansprüche, welche in der Methode selbst liegen. Für diese ist keineswegs *ein* Forscherindividuum, ein einzelnes Subjekt, allein verantwortlich: weder für die Generierung der Ansprüche noch für ihre (Nicht-)Erfüllung. In diesem Sinn gilt: *Der Fokus auf die Methode kollektiviert die forschenden Subjekte*. Den intern politisierenden Anspruch einer philosophischen Methode zu untersuchen, macht Kritik ein gutes Stück weit vom individuellen Habitus wie auch von Idiosynkrasien einzelner Forscherpersönlichkeiten unabhängig. Der vorliegende Essay richtet sich folglich nicht darauf, ob und in welchem Ausmaß Husserl oder andere Phänomenologinnen offen und empfänglich für Kritik waren, ob und in welchem Ausmaß sie sich um Selbstkritik bemüht haben. Zweifellos sind das interessante Fragen. Hier geht es jedoch darum auszumachen, was im Spielraum der Methode als solcher und prinzipiell liegt; wie Art, Richtung und Niveau der Kritik zu bestimmen sind, welche in der *Ratio* der betreffenden Methode selbst liegen.^39^

## Chancen und Gefahren einer Politisierung der Phänomenologie

Phänomenologische Beschreibungen und Analysen extern zu politisieren, ist nicht per se problematisch. Im Gegenteil: Im Sinne einer lebendigen Diskurskultur sind externe Politisierungen wünschenswert. Sie sind überdies, je nach Themenfeld, häufig erwartbar. Veranlasst werden sie meist dadurch, dass sich ein und dieselbe Sache *von verschiedenen realen Subjektpositionen bzw. Standorten* verschieden darstellt. Wird die betreffende Ansicht in einer öffentlichen Debatte reklamiert und im Zuge dessen der Anspruch erhoben, dass die zugehörigen Urteile in der Sache selbst begründet und mittels Hinweis auf Evidenzen und Argumente ausweisbar sind, ist externe Politisierung jedoch zugleich an die „interne“  Struktur einer bestimmten Idee der Vernunft gebunden. Auch wenn diese in verschiedener Weise expliziert werden kann, ist die Rückbindung an die Idee der Vernunft insofern entscheidend, als infolgedessen Standortbekundungen und standortabhängige Erscheinungsweisen, Ideen, Thesen u. dgl. nicht *eo ipso* als Geltungsbegründung verstanden sind. Sie werden vielmehr im Bewusstsein geäußert, dass sie mit Hilfe eines standortüberschreitenden Standards der Vernünftigkeit artikuliert und diesem gemäß in ihrem Geltungsanspruch verteidigt werden müssen. Dass ein Standard standortüberschreitend ist, schließt eine Einschränkung seines Geltungsanspruches auf den jeweiligen, einzelnen Standort aus, verlangt jedoch nicht, dass der Beurteilungsstandard prinzipiell standortneutral—jenseits aller möglichen Standorte—zu verankern wäre. Diesbezüglich ist die Differenz zwischen einem *view from nowhere* und einem *view from here* zu beachten,^40^ welche auch dann unaufhebbar ist, wenn ersterer als ein bloß methodisches Telos, als ein Näherungsziel, konzipiert ist. Die oben entwickelte Argumentation^41^ anerkennt die Unaufhebbarkeit dieser Differenz; sie formuliert ein dynamisches Moment des *In-Between* zwischen *view from nowhere* und *view from here*: Multiperspektivismus bzw. Standpunktpluralismus legen den methodischen Habitus der Phänomenologie auf einen *view from elsewhere* fest.

Der Vorteil eines extern *und* intern politisierten Denkens—wenn man will: einer gemischten Politisierung—liegt darin, dass die Bewährung phänomenologischer Problemformulierungen und Analysen auf eine breitere Basis gestellt wird. So können Sichtbeschränkungen und Irrtümer, die sich dem unbemerkten Einwirken von Vorurteilen oder Bias-Strukturen auf Seiten der phänomenologisierenden Subjekte verdanken, entdeckt und korrigiert werden.^42^ Politisierungen solcher Art als eine Erkenntnischance zu verstehen,^43^ entspricht der Einsicht, dass die philosophierenden Subjekte faktisch verschiedenen sozialen Gruppen, Milieus, Ethnien und Kulturen angehören, dass sie verschiedene Gender-Identitäten entwickeln usw. Entsprechend komplex stellt sich die Frage nach offenkundigen und verborgenen Standortabhängigkeiten dar. Dies gilt umso mehr als die handelnden Subjekte sich selbst nicht bzw. nicht vollständig transparent sind. Ebenso wie Husserl dies für die phänomenologische Forschungsarbeit im Allgemeinen moniert, ist auch die Selbstaufklärung individuell-standortbedingter Vorurteile und sonstiger Interpretamente, welche auf soziale Herkünfte, Bindungen und Identitäten zurückgehen, ein Anspruch, der nur kooperativ realisiert werden kann: als Gemeinschaftsleistung einer Gruppe von (selbst)kritischen Denkerinnen.

Worin liegen dem gegenüber nun die Gefahren einer externen Politisierung des Denkens?^44^ Das Vorliegende ist ein Plädoyer dafür, diese Debatte primär im Lichte der Frage zu führen, was Politisierung mit der Befähigung zu vernünftigem Denken zu tun hat und was dies auf Seiten der denkenden Subjekte und ihres Selbstverhältnisses erfordert. Fähigkeiten und die zu ihrer Entwicklung bzw. Aufrechterhaltung notwendigen Erfordernisse zu benennen, zielt darauf, die Chancen einer Politisierung der Phänomenologie zu erfassen und sie infolgedessen bewusst in Anspruch zu nehmen und zu gestalten. Wird dagegen von den Gefahren einer Politisierung gesprochen, so bezieht sich dies darauf, dass interne und externe Politisierung in ein derartiges Spannungsverhältnis zueinander geraten können, dass es unmöglich ist, die beiderseitigen Ansprüche aufrechtzuerhalten. Der Fall, der hier interessiert, ist der Hegemonieanspruch radikaler externer Politisierung im oben eingeführten Sinn. Genauer interessiert die Frage nach dessen Grenzen: Welchen Restriktionen müssen externe Politisierungsansprüche unterliegen, damit ihre Realisierung nicht die Möglichkeit vernünftigen Denkens unterminiert?^45^

Häufig ist der Ausgangspunkt kritischer Befragungen der entgegengesetzte. Dies ist der Fall, wenn ein philosophisches Denken als suspekt erscheint, weil es sich im Sinne politischer oder weltanschaulicher Positionierungen als neutral bzw. indifferent und in diesem Sinne als apolitisch darstellt. Dabei geht es um konkrete Gedankeninhalte, um ein material gebundenes Denken. Dem gegenüber interessiert hier Politisierung im Sinne der Ermöglichung bzw. Befähigung oder aber der Verhinderung und Unterminierung vernünftigen Denkens, welches als solches Begründungsansprüche reklamiert, die einzelne Anwendungen oder Instanziierungen transzendieren. Allein als Träger derartiger Begründungsansprüche kann vernünftiges Denken als ein im ursprünglichen Wortsinn kritisches Denken auftreten: als eines, das zu relevanten Differenzierungen imstande ist und diese unter steter Orientierung am Erkenntnis- und Wahrheitsziel ausübt—mit Bezug auf welche Gegenstandsbereiche immer. Ist die zugehörige Einstellung des Strebens nach Differenzierung und Klarheit, die die Denkende zu dem einnimmt, worüber sie denkt wie auch zu sich selbst als Denkender, indifferent gegenüber wechselnden Materien des Denkens, so verweist dies darauf, dass die betreffenden Begründungsstandards rein formale sind. Als solche betreffen sie alle Arten von Gegenständen gleichermaßen. Begründungsstandards und -ansprüche differenzieren sich jedoch auch am gegebenen Erfahrungsmaterial entsprechend verschiedener Gegenstandsbereiche.^46^ Inhärente (formale oder materiale) Normierungs- bzw. Begründungsansprüche des Denkens müssen konkret zur Anwendung gebracht und an *der Sache selbst* erprobt werden. Das schließt das Austragen von Unstimmigkeiten und Konflikten auf Basis pluraler epistemischer, evaluativer und volitiver Perspektiven im Sinne sukzessiver Klärung und kontroverser Urteilsbildung über bestimmte Gegenständlichkeiten ein.^47^ Die Art und Weise, in der Sprache in derartigen Klärungsprozessen verwendet wird, ist mit Blick auf Politisierungen hochsensitiv. Das bedeutet u. a., dass das, was sich als unmittelbar gegeben darstellt, nicht nur methodisch vermittelt, sondern auch sprachlich in komplexer Weise normativ infiltriert sein kann.

## Autonomie der Vernunft: warum sie mit radikaler externer Politisierung inkompatibel ist

Oben wurde festgestellt, dass die Gefahren einer Politisierung des Denkens darin gründen, dass interne und externe Politisierung konfligieren und inkompatibel sein können. Was heißt das genau? Ein starker Hegemonieanspruch externer Politisierung eliminiert vernünftiges und kritisches Denken zugunsten gesellschaftlicher Positions- und Durchsetzungskämpfe. Ist dies der Fall, werden inhärente (formale und materiale) Normierungs- bzw. Begründungsansprüche des Denkens durch die sogenannte *normative Kraft des Faktischen* ersetzt. Letztere liegt auf Seiten derer, die die stärkste Handlungs- und Durchsetzungsmacht in einem öffentlichen Meinungsgeschehen haben.^48^ Handlungs- und Durchsetzungsmacht einerseits und sachbezogene Einsicht andererseits können zufällig koinzidieren. Erfahrungsgemäß ist das häufig nicht der Fall, und wenn es der Fall ist, bleibt beides nach Idee und Natur geschieden. Denn Handlungs- bzw. Durchsetzungsmacht gibt per se keine Evidenz für das Vorliegen einer sachbezogenen und sachgebundenen Einsicht; sie ist etwas durchaus verschiedenes. Soziale Autorität und epistemische Autorität können zwar kontingenter Weise in „Personalunion“ auftreten, stellen jedoch, für sich betrachtet, verschiedene Arten von Autorität dar, denen unterschiedliche Interessen, Befähigungen, Expertisen und Wirksamkeiten zugrunde liegen.^49^

Ein diesbezügliches Beurteilungsprinzip kann lauten: Ein Ungleichgewicht im Sinne der Prävalenz externer Politisierung und, entsprechend, eines Vorherrschens sozialer Autorität führt zur Aufhebung vernünftigen Denkens und, entsprechend, dazu, dass Subjekte den Status und Anspruch epistemischer Autorität verlieren bzw. die Zuschreibungsbedingungen mit Bezug auf letztere unsicher werden.^50^ Welche konkreten Standortbindungen auch immer mittels einer radikalen externen Politisierung vertreten werden, entscheidend ist, dass ihre Verteidigerinnen dann nicht mehr den Anspruch erheben können, die zugehörigen Standpunkte begründen, das heißt qua Denken (sc. begrifflichem Unterscheiden, logisch-folgerichtigem Argumentieren, Mittel-Zweck-Räsonnement, Deliberation mit Blick auf variable Zwecksetzungen u. dgl.) rechtfertigen zu können. In dieser radikalen Form auftretend, ist eine externe Politisierung des Denkens selbstaufhebend: sie verunmöglicht die Begründung (Rechtfertigung) der eigenen Kritik wie auch generell jede kritische Standpunktprüfung, indem sie soziale Autorität und Macht praktiziert und damit die Verzichtbarkeit epistemischer Autorität suggeriert. Dieses Verhalten legt im Hinblick auf interne Begründungsansprüche (wie immer diese im Einzelnen formuliert sind) zwei Interpretationen nahe: Entweder negiert eine radikal externe Politisierung des Denkens stillschweigend die eigene Fallibilität, indem sie selbstverständlich Gewissheit für das Behauptete in Anspruch nimmt. Oder sie stipuliert überhaupt die Nichtigkeit, die Irrelevanz sachbezogener (immanent regulierter) Begründungsansprüche. Gemäß beiden Interpretationen, die auch in wechselseitiger Verstärkung gemeinsam praktiziert werden können, disqualifiziert sich eine radikale externe Politisierung des Denkens als vernünftiges Denken. Denken wird zu einem bloßen Pseudo-Denken, das sich über die Selbstgewissheit und Wirksamkeit sozialer Durchsetzungsmacht in einem Kreislauf der Selbstbestätigung bewegt—und damit den Titel „Denken“ nicht mehr verdient. Sofern das solcherart politisierte „Denken“ anderes sein wollte als ein mehr oder weniger sophistiziertes *Überleben des Stärkeren,* greift das, was ich das „Paradoxon der externen Politisierung“ nenne: Je weiter sich soziale Durchsetzungsmacht steigert, je umfassender sie de facto ausgeübt und je stärker sie wird, desto schwächer wird sie epistemisch; desto offenkundiger die Einbuße an intellektueller Kraft und Ernsthaftigkeit.

Vom internen Standpunkt des Denkens lässt sich das Problem wie folgt erläutern. Welche Art von Gedanke wäre es, wenn ein Subjekt sich selbst als in seinem Denken vollständig bestimmt denken müsste von Inhalten, Ideen und Auffassungen, welche ihm extern—von sozial Einflussreichen, von Meinungsführern und Mächtigen—oktroyiert würden?^51^ Könnte solcherart Aufgenötigtes und Erzwungenes überhaupt als ein Gedanke, als ein möglicher Inhalt des Denkens verstanden werden? Diese Frage findet sich in nahezu gleicher Form in einem Essay Thomas Nagels mit dem Titel „Why We Can’t Understand Thought from the Outside“.^52^ Nagel formuliert hier Erwägungen und Thesen, die weitgehend mit Husserls Argumentation zur Widerlegung des Psychologismus in der Logik übereinstimmen. Die entsprechenden Ausführungen finden sich in Husserls *Prolegomena zur reinen Logik* von 1900 und, in einer argumentativen Variation, in seinen späten Ethik-Vorlesungen in den 1920er Jahren als Kritik des Hedonismus und Subjektivismus in der Ethik. Nagels strukturell ähnliche Kritik lautet in aller Kürze zusammengefasst wie folgt.^53^ Nagels initiale Frage in dem oben genannten Essay setzt bei der Überlegung an, dass wir versuchen können, eine externe psychologische (oder, wahlweise, soziologische, biologische, hirnphysiologische etc.) Kritik unserer selbst gedanklich bis zur äußersten Grenze voranzutreiben. Das hieße: die Externalisierung—die externe Betrachtung unserer selbst—als vollständig durchgeführt vorzustellen, mithin uns selbst als denkende Subjekte vollständig in Erkenntnisobjekte verwandelt zu denken:

However, the pursuit of self-awareness breaks down if we try to extend this kind of external psychological criticism of ourselves to the limit—which must happen if we entertain the possibility that nothing in human thought really qualifies as reason in the strong sense I wish to defend. For we are then supposed to consider the completely general possibility that there are contingent and local explanations of the sources of all our convictions, explanations that do not provide justifications as strong as reason would provide, if there were such a thing. And the question is, what kind of thought is this? It purports to be a view of ourselves from outside, as creatures subject to various psychological influences and prey to certain habits, but what are we supposed to be relying on in ourselves to form that view? (Nagel, 1997, 14)

Zur Erklärung: „Vernunft im emphatischen Sinne“ (*reason in the strong sense*) bezieht sich hier auf ein Vermögen des Denkens, das universelle Anwendbarkeit und Gültigkeit besitzt und imstande ist, sämtliche Urteile zu überprüfen und gegebenenfalls als begründet auszuweisen. Auch wenn nicht auszuschließen ist, dass externe Kritiken und Beeinspruchungen gegen immanente Vernunftansprüche *immer wieder in Einzelfällen* erfolgreich sind in dem Sinn, dass der Nachweis geführt werden kann, dass standortabhängige Restriktionen und Verzerrungen irrtümlich mit Objektivitätsansprüchen versehen wurden, so ist doch notwendig zweierlei der Fall: Erstens, jede solche Kritik und Beeinspruchung unterliegt selbst dem Anspruch, begründet bzw. begründbar zu sein. Begründungen nehmen jedoch Vernunftleistungen in Anspruch. Deshalb kann eine standortabhängige Kritik der Vernunft, *sofern sie selbst den Anspruch erhebt, vernünftig zu sein*, nie eine absolute oder totale sein, welche sämtliche Bereiche der Vernunft umfasste.^54^ Zweitens, sobald externe Kritiken und Politisierungen des Denkens als radikale, die Vernunft in ihrer Gesamtheit umfassende und entwertende auftreten, unterminieren sie sich selbst in ihrem Anspruch, eine relevante Kritik und ein „besseres“, richtigeres Denken zu repräsentieren.

Denken ist nur dann wahrhaft Denken, wenn die Autonomie der Vernunft gegenüber externen Politisierungen und sozialen Machtausübungen verschiedener Art gewahrt bleibt. Eine Schwierigkeit liegt jedoch in Folgendem. Gedanklich gibt es kein Mittleres zwischen vernünftig denken und aufgrund von Macht- oder Gewaltausübung zu bestimmten Positionierungen genötigt werden (und sich selbst als so Genötigte verstehen). Entweder wird epistemische Autorität ausgeübt und von anderen zugestanden oder dies ist nicht der Fall. Unter realen Bedingungen des Lebens und Denkens finden sich denkende Subjekte, auch aufgrund ihrer beschränkten Selbsttransparenz, jedoch häufig in einem Graubereich. Es ist nicht der Fall, dass jeder jederzeit klar wäre, ob sich die Resultate ihrer Überlegungen dem eigenen Denken und der eigenen Urteilsfähigkeit verdanken oder ob diese zumindest partiell korrumpiert sind durch die latente Einwirkung und Beeindruckung durch soziale Manipulation und Machtausübung. Auch hier gilt wie oben in anderem Kontext festgestellt, dass individuelle Selbsterforschung und Selbsterkenntnis auch von Art und Niveau einer gemeinschaftlich praktizierten Denkkultur abhängig sind. Darüber hinaus ist jedoch ein Argument Nagels zu bedenken, das sich, soweit ich sehe, nicht entkräften bzw. nicht als falsch erweisen lässt, ohne dass es in der Selbstanwendung zur Selbstaufhebung des Denkens käme. Das Argument lautet: Es gibt eine Grenze jeder möglichen extern induzierten Korruption des eigenen Denkens, die wir mit Bestimmtheit feststellen können—nämlich: Die Behauptung, dass unser Denken durch äußere Einflüsse und Determinationen—bzw. in meiner Terminologie: durch radikale externe Politisierung—*vollständig* korrumpiert wäre, ist ein Gedanke, der keiner ist, weil er nicht denkmöglich ist. Dieser Gedanke kann in keinem für uns verständlichen Denken vollzogen werden, ohne sich selbst aufzuheben.^55^ Das ist eine absolute Grenze der Vernunftkritik, die wir auch im Denken über uns selbst, in unserem Selbstverhältnis, nicht überschreiten können (als die Lebewesen, die wir sind).

Im vorliegenden phänomenologischen Rahmen, Bezug nehmend auf die oben ausgeführten Implikationen einer methodisch geformten Subjektivität und einer entsprechend grundgelegten Ethik der Überzeugungsbildung, kann eine fundamentale Schwierigkeit bearbeitet werden, die Nagel so formuliert: „To be rational we have to take responsibility for our thoughts while denying that they are just expressions of our point of view. The difficulty is to form a conception of ourselves that makes sense of this claim.“ (Nagel, 1997, 11) Die phänomenologische Antwort auf diese Frage liegt in dem Verständnis, dass einerseits die Erste-Person-Perspektive (qua Intentionalität) ein unaufhebbares Strukturmoment jeder möglichen Weltbeziehung darstellt, andererseits die oben entwickelten Ansprüche einer internen Politisierung der phänomenologischen Methode dahingehen, sich in jeder Untersuchung konkreter Phänomene oder Probleme einer partiellen Entindividualisierung und Selbstüberschreitung zu unterstellen. Denn anders sind die Ansprüche der Vernunft auf extensive und intensive Exploration dessen, was jeweils erfahren ist, und auf bestmögliche Prüfung gegebener Evidenzen nicht realisierbar. Das heißt: Das von Thomas Nagel prägnant benannte Problem findet eine zumindest partielle Antwort in der Konzeption eines erweiterten Selbst. Gemäß der von der Idee der Vernunft selbst geforderten Multiperspektivität ihrer Realisierung muss ich anerkennen, dass zwar ich es bin, an deren konkreter Vernunftausübung die Verwirklichung von Vernunft im Einzelfall hängt, dass Vernunftausübung gleichzeitig aber heißt, meine individuelle Perspektive zu überschreiten bzw. immer schon überschritten zu haben. Als vernünftiges Subjekt bin ich per se ein erweitertes Selbst. Ein im Sinne der obigen Überlegungen konzipiertes erweitertes Selbst verlangt ein entsprechend erweitertes Verständnis von kollektiver Verantwortung für Gedanken und Urteile—und generell für jede Art der Vernunftausübung.^56^

Der prioritäre Status von Intersubjektivität gegenüber einem methodisch isolierten und meist unter einem Generalverdacht des Cartesianismus stehenden einzelnen Subjekt stellt ein Hauptanliegen der neueren *critical phenomenology* und ihrer Abgrenzung von der klassischen Phänomenologie, insbesondere jener Husserls, dar. Deshalb will ich abschließend zumindest andeuten, wie sich der oben entwickelte Gedankengang zu dieser neuen Formation in der Theorielandschaft verhält. Meine Überlegungen widersetzen sich einer dichotomischen Betrachtung von klassischer und kritischer Phänomenologie.^57^ Sie verstehen sich aber durchaus als ein Plädoyer zugunsten einer sorgfältigen Re-Vision der phänomenologischen Tradition im Lichte aktueller Interessenlagen. Das schließt inhaltliche Positionierungen und Konzepte ebenso ein wie methodologische Grundlagen und bislang unausgeschöpftes Anwendungspotential. Mit Blick auf diese Spannweite systematischer Referenz stimmt die obige Argumentation mit jenen Darstellungen überein, welche den kritischen Anspruch der klassischen Phänomenologie und die Kontinuität von klassischer und kritischer Phänomenologie betonen—bei gleichzeitiger Skepsis hinsichtlich der Bezeichnung „kritische Phänomenologie“, die der klassischen Phänomenologie implizit jeden kritischen Charakter abspricht.^58^ Was dennoch aus Sicht der obigen Analyse gegen eine von der Kontinuitätsthese nahegelegte integrative Sichtweise spricht, wonach eine umfängliche methodische bzw. methodologische Kompatibilität von klassischer (v. a. Husserlscher) und kritischer Phänomenologie bestünde, ist der von Seiten der letzteren erhobene post-Foucault’sche Anspruch politischen Aktivismus.^59^ Dieser Anspruch verlangt eine weitergehende Analyse und unterliegt jedenfalls dem oben skizzierten Selbstaufhebungsargument. Das heißt: Wenn unter „critical phenomenology“ eine Phänomenologie zu verstehen ist „that mobilizes phenomenological description in the service of a reflexive inquiry into how power relations structure experience as well as our ability to analyze tha experience“ (Weiß, Murphy & Salamon 2020, xiv), dann muss der operativen Reflexivität epistemische Autorität insofern zukommen, als die externe Determination durch Machtverhältnisse, die sowohl auf der gegenständlichen als auch auf der Meta-Ebene der Gegenstandsanalyse festgestellt wird, nicht als umfassend gedacht sein kann.^60^ Andernfalls vernichtete kritisch-phänomenologisches Denken sich selbst als Denken.

Der oben entwickelte Vorschlag, wie über die Politisierung der Phänomenologie in differenzierter Weise gedacht werden kann, versteht sich auch als ein Rahmen für die weiterführende Analyse des möglichen Spielraums einer kritischen Phänomenologie. Wie die Konzeption des Perspektivenpluralismus und des erweiterten Selbst verdeutlicht, ist insbesondere Intersubjektivität immer schon integraler Bestandteil einer im Geiste Husserls verstandenen klassisch-phänomenologischen Methode. Es wäre jedoch ein Irrtum zu meinen, dass damit die grundlegende Bedeutung und Rolle der Ersten-Person-Perspektive in Frage gestellt wäre. Im Gegenteil: Ohne ein aus eigener Erfahrung Vertrautsein mit der Ersten-Person-Perspektive bliebe unverständlich, was es heißt, die Welt, die anderen und (in Vergegenwärtigungen vergangener Erlebnisse) uns selbst perspektivisch gegeben zu haben. Ebenso bliebe ohne die Verankerung im je eigenen originären Erleben unverständlich, was es heißt, ein Selbst zu sein. Allein unter der Bedingung der Ersten-Person-Perspektive macht die Rede von „Erweiterung“ Sinn—ungeachtet dessen, dass diese Rede den rekonstruktiv-analytischen Standpunkt einer phänomenologischen Methodenlehre einnimmt. Das bedeutet u. a., dass keineswegs bestritten wird, dass in der lebensweltlich und ontogenetisch erfahrenen Realität der Weg der Selbstfindung umgekehrt verläuft: von einem immer schon sozial erweiterten Selbst mittels Reflexion zurück zur Entdeckung meines individuellen Selbst und biografisch-intimen Ich. Hier bestätigt sich, dass ein umfassendes und tiefes Verständnis der phänomenologischen Methode (z. B. im Hinblick auf die verschiedenen Typen der Reduktion, die Einstellungslehre, die Stratifizierung des Bewusstseins und die Schichtenanalyse) grundlegend ist, um einzelne Aussagen wie auch Analysen spezifischer Phänomene bzw. von Teilaspekten einer komplexen Struktur von Subjektivität richtig einordnen und interpretieren zu können.

## Fazit: Wie Gefahren vermeiden und Chancen nützen?

Meine Ausgangsfrage („Ist das Gegebene noch zu retten?“) war aus rhetorischen Gründen verkürzt gestellt. Die eigentliche und vollständige Frage lautet: Ist eine phänomenologische Intentionalanalyse, welche auf die Gegebenheit des Gegebenen fokussiert, im Lichte der rezenten Forderung nach einer Politisierung des Denkens noch legitim und sinnvoll? Die Antwort, die aus Obigem resultiert, ist: Ja, sofern Phänomenologinnen in der Nachfolge Husserls eine Zickzack-Methode praktizieren, die es erlaubt, auch kritische Einwände, die in externen Politisierungen des Denkens gründen, aufzunehmen und im Hin und Her zwischen gegenständlicher Ebene der Phänomenanalysen einerseits und Reflexion der dabei zur Anwendung gebrachten Methode und Begrifflichkeit andererseits produktiv zu verwerten. Mit Bezug auf die Revision und Verbesserung der Begriffe und Kategorisierungen, aber auch der Methoden, muss die phänomenologische Untersuchung im Dienste des sie leitenden Erkenntnis- und Wahrheitsinteresses für interne und externe Kritik durchlässig sein. Externe Kritik unterliegt jedoch Grenzen.

Über die Legitimität und Sinnhaftigkeit einer phänomenologischen Untersuchung im Allgemeinen wird auf Basis immanenter normativer Standards und Ansprüche entschieden—und kann nicht anders entschieden werden. Auch wenn Revisionen aufgrund extern politisierender Kritiken mit Bezug auf die phänomenologische Beschreibungs- und Analysearbeit nicht ausgeschlossen sind, ist diesen dennoch eine definitive Grenze gesetzt: Die fragliche Politisierung darf nicht zu einer Selbstaufhebung des Denkens zugunsten sozialer Positionierungs- und Durchsetzungsmacht führen. Eine derart radikale externe Politisierung ist mit der Autonomie bzw. Autorität der Vernunft, wie sie phänomenologische Forschung im Geiste Husserls und anderer Denkerinnen in Anspruch nimmt, unverträglich. Wenn wir nicht hinter die intellektuellen Standards einer phänomenologischen Erfahrungsanalyse zurückfallen wollen, müssen wir allerdings darauf achten, interne und externe Politisierung nicht starr, dogmatisch und exklusiv einander entgegenzusetzen. Sofern externe Politisierung nicht als radikale Aufhebung des Denkens auftritt, muss eine gewisse Durchlässigkeit von innen nach außen und vice versa gewährleistet sein. Wie stellt sich diese Durchlässigkeit dar und wie ist sie begründet?

*Von innen nach außen*: Wenn im internen Überzeugungsbildungsprozess sachlich relevante Gesichtspunkte nicht bzw. nicht in gebührendem Ausmaß berücksichtigt werden, weil sich diese Gesichtspunkte insbesondere jenen aufdrängen, die bestimmte soziale Milieus, kulturelle Praxen, Ethnien, Genderrollen etc. aus eigener Erfahrung kennen, diese Subjekte aber in der internen Argumentation keine Stimme haben oder als Minderheit nicht gleichgewichtig zu Wort kommen, dann muss eine interne Debatte über Inklusion vs. Exklusion geführt werden und/oder eine Öffnung nach außen erfolgen, um die interne Diskurspraxis entsprechend zu erweitern bzw. zu korrigieren. Zu fordern und unverzichtbar ist das aber nicht um der vollständigen Abbildung einer gegebenen sozialen und kulturellen Realität willen, sondern um einer sachgerechten Überzeugungsbildung und Problemanalyse willen.^61^

*Von außen nach innen*: Soll eine gesellschaftliche Debatte den Anspruch erheben können, rationalen Standards zu folgen und entsprechend zu Ergebnissen zu führen, die *so weit als möglich* von standpunktbedingten Einschätzungen und Präferenzen frei sind, dann ist dafür Sorge zu tragen, dass die Teilnehmerinnen an öffentlichen Debatten wissenschaftlicher und philosophischer Expertise zugänglich und dieser gegenüber offen sind. Dies erfordert, öffentliche Meinung und kulturelle Bildung dahingehend zu stärken, dass Tendenzen zu Wissenschafts- bzw. Theorieskepsis und Experten-Bashing entschieden und effektiv entgegengesteuert wird—im Bewusstsein, dass das Niveau der öffentlichen Meinungsbildung und in der Folge die Demokratiefähigkeit der Bürgerinnen daran hängt, dass zwischen begründeter Argumentation und sozialer Durchsetzungsmacht unterschieden und dieser Unterschied individuell nachvollziehbar gemacht wird.

Durchlässigkeit im Sinne eines beidseitig offenen Austausches zwischen öffentlicher Meinungsbildung und philosophischer bzw. wissenschaftlicher Theoriebildung zu befördern, heißt *nicht*, einer latenten und intransparenten Vermengung von Epistemologie mit (tages-)politischen Interessen zuzustimmen. Ebenso heißt es *nicht*, die irrige Auffassung zu vertreten, dass Politik im herkömmlichen Sinn weltanschaulich gebundener Interessens- und Standpunktvertretung durch phänomenologische Problemanalysen oder durch wissenschaftliche Forschung zu ersetzen oder auf diese zu reduzieren wäre.^62^ Hier wie so oft verlangt Klarheit des Denkens, bestehende Differenzen anzuerkennen. Was ist das Fazit der obigen Überlegungen im Hinblick auf eine differenzierte Rede von „Politisierung“?

Eine philosophische Konzeption der Politisierung kann es nicht dabei belassen, interne und externe Ansprüche und Konzepte explizit zu machen und miteinander zu konfrontieren. Sie muss zumindest ein Stück weit das Verhältnis von interner und externer Politisierung aufklären. Der obige Versuch, dieser Aufgabe nachzukommen, bringt eine semantische Transformation und äquivoke Verwendung des Terminus „Politisierung“ mit sich. Hier gilt, was Quentin Skinner generell für Begriffswandel festhält: „[T]hat the application of a term with a new range of reference may eventually put pressure on the criteria for applying it.“ (Skinner, 1989, 15) Im vorliegenden Fall gehen die betreffenden Änderungen mit der Einsicht einher, dass die stillschweigende Dichotomisierung, die einer weit verbreiteten Verwendung von „Politisierung“ zugrunde liegt—dass entweder ein politisches Engagement und Bekenntnis im gewöhnlichen Verständnis alltäglicher Realpolitik vorliege oder aber eine lebensferne generelle Abstinenz bzw. Ignoranz mit Bezug auf Fragen der Politisierung—fragwürdig und zurückzuweisen ist. Demgemäß unterliegt etwa (um einen konkreten Anwendungsfall zu nennen) die identitätspolitische Revision und Neubewertung der klassischen Phänomenologie^63^ nicht nur dem Anspruch, ihr zugrunde liegendes Verständnis von „Politik“ und „Politisierung“ explizit zu machen. Sie unterliegt auch dem Anspruch, sich mit alternativen Konzepten von Politisierung auseinanderzusetzen, welche im Rahmen eben jener Theorie(traditione)n entwickelt werden, deren Ungenügen mit Hilfe eines (im engeren Sinn) politischen Konzepts von Politisierung erwiesen werden sollte. Mit anderen Worten: Ob die Debatte über eine zu fordernde Politisierung des Denkens selbst eine im engeren Sinn politische Debatte ist oder nicht, ist Teil des Problems. Ein diesbezüglich vorweg eingenommener und im engeren Sinn politischer Standpunkt kann nicht als Lösung des Problems präsentiert werden. So vorzugehen entspräche einer politisch motivierten (und sanktionierten) Beendigung der Debatte—auch dann, wenn dies unausgesprochen bliebe und durch eine Fortsetzung des Diskurses verschleiert würde. Die Debatte tatsächlich und in der vollen Reichweite des Problems zu führen, bedeutet anzuerkennen, dass sie auch darüber geführt wird, welche Verwendungen des Terminus „Politisierung“ möglich, legitim und sinnvoll sind. Dies schließt in weiterer Folge die diskursive Erörterung der produktiven oder disruptiven, erkenntnisförderlichen oder -hemmenden Folgen von Politisierungen ebenso ein wie etwa die Bestimmung wertender Konnotationen der Rede von „Politisierung“.

Soweit der vorliegende Essay die Idee einer Politisierung philosophischer Theorie(bildung) bzw., speziell, einer *politischen Konstitution der Phänomenologie* legitimiert, bezieht sich dies auf die Art und Weise, wie das Streben nach Erkenntnis, auch mit Blick auf seine potenziell gesellschaftskritische und -politische Relevanz hin, konzipiert und kultiviert wird. Das heißt: wie ein bestimmter Umgang mit Phänomenen, Evidenzen und Intentionalanalysen entwickelt, als eine Erkenntnispraxis instituiert und überprüft wird, wie zweckdienliche Methoden und Begrifflichkeiten etabliert werden und wie sich die an dieser Konzeptualisierungs- und Analysearbeit beteiligten Subjekte zu einer Erkenntnisgemeinschaft—einer *Polis* der geistig und erfahrend Tätigen—formieren.^64^ Das schließt die Möglichkeit und Wirklichkeit von Dissens und die Verpflichtung zum „zivilisierten“ Austrag konfligierender Auffassungen auf Schritt und Tritt ein. Mit tagespolitischen Interessen, weltanschaulichen Bindungen und den daraus resultierenden Antagonismen hat dies nichts zu tun. Umgekehrt, im Sinne der Phänomenologie von einer *philosophischen Konstitution der Politik* zu sprechen, ist nur soweit berechtigt, als sich die Politik Treibenden in ihren inhaltlichen Stellungnahmen und Sichtweisen einem Reflexions- und Selbstaufklärungsanspruch unterstellen, der weder einem parteipolitischen Räsonnement noch dem gesellschaftlichen Durchsetzungsinteresse einer Weltanschauungsgemeinschaft bzw. einer pragmatisch geeinten Allianz von Nutznießern untergeordnet wird. Das heißt: Politik ist nur soweit philosophisch konstituiert, als sie sich über Parteigrenzen und Parteiinteressen hinweg eine Rechenschaftspflicht vor allen Vernünftigen auferlegt, die nach Gründen und Begründungen fragen. Philosophische Konstitution, so verstanden, bleibt ein widerstrebendes, störendes Moment in der Politik—sofern diese im gewöhnlichen, alltäglich wirksamen Sinn verstanden ist. Politische Konstitution der Philosophie, andererseits, erscheint als ein integrales Moment der Phänomenologie—sofern wir dem oben entwickelten Verständnis von interner Politisierung Folge leisten. Liegt dagegen die gewöhnliche Auffassung von Politik und Politisierung zugrunde, sind Philosophie und Politik unverträglich. Denn ein philosophischer Anspruch an Denken und Argumentieren, an den Umgang mit Erfahrungen und Erfahrungssubjekten entzieht sich der Autorisierung durch und der Verpflichtung auf Parteiinteressen.

Um am Ende noch einmal an den Untertitel der vorliegenden Erörterung anzuknüpfen: Wie können wir die Gefahren einer Politisierung der Phänomenologie vermeiden und die darin liegenden Chancen nützen? Wir vermeiden die Gefahren—insbesondere die der Selbstaufhebung des Denkens—indem wir zwischen verschiedenen (internen und externen, auf der Gegenstandsebene und auf der Metaebene ansetzenden) Formen und graduellen Ausprägungen einer Politisierung unterscheiden. Wir vermeiden die Gefahren, indem wir diese verschiedenen Formen und Gradualitäten analysieren und verdeutlichen, dass radikale externe Politisierung zur Selbstaufhebung vernünftigen Denkens führt, während interne Politisierung und nicht-radikale externe Politisierung philosophische und wissenschaftliche Forschung nicht prinzipiell in Frage stellen, sich vielmehr in verschiedener Hinsicht als wichtig und nützlich zur Verbesserung der betreffenden Erkenntnisvorhaben erweisen können.

Die Chancen einer so verstandenen Politisierung der Phänomenologie können auf verschiedenen Ebenen konkretisiert werden. Der Gesichtspunkt der Politisierung sollte, *erstens*, als immanente (und habitualisierte) Anforderung an den Gebrauch phänomenologischer Verstehens- und Analysemethoden praktiziert werden; er sollte, *zweitens*, Bestandteil der methodologischen und theorierahmenbezogenen Selbstreflexion sein und damit Anteil an der Selbstkritik phänomenologischen Philosophierens gewinnen. Der Nutzen, der darin liegt, die Politisierung der Phänomenologie sowohl im Methodengebrauch als auch in dessen Meta-Reflexion vor Augen zu haben, ist freilich nicht auf die Verbesserung phänomenologischer Forschung beschränkt. Daraus könnte darüber hinaus in verschiedenen Untersuchungsfeldern, *drittens*, eine Phänomenologie des Lernens aus Erfahrung—etwa mit Bezug auf ästhetische Erfahrung und andere Arten von Werterfahrung, mit Bezug auf historische und interkulturelle Erfahrung, intersektionale Erfahrung—entwickelt werden, welche auf die transzendentale Dimension der Gegebenheitsweisen des Gegebenen abzielte. Kernthema dieser Konzeption des Lernens wäre der (selbst)kritische Umgang mit der im gesellschaftlichen Alltag allerorts anzutreffenden Pluralität von Gegebenheitsweisen und den daraus resultierenden Meinungsverschiedenheiten. Eine diesbezügliche Untersuchungsmaxime könnte lauten: Je genauer, detaillierter und umfassender mögliche Wahrnehmungs- und Beurteilungsstandorte und deren epistemische Implikationen untersucht werden, desto differenzierter können die Gründe für bestehende Meinungsverschiedenheiten benannt werden. Die Zielsetzung wäre, einen weitestmöglich vorurteilsfreien, auf rationaler Urteilsbildung basierenden Umgang mit Meinungsverschiedenheiten zu etablieren. Dies wäre ein wichtiger und grundlegender Schritt in der Kultivierung kritischen Denkens wie auch demokratischer Umgangsformen.

Der Gesichtspunkt der Politisierung sollte, *viertens*, die Entwicklung einer Ethik des phänomenologischen Denkens motivieren, die nicht nur nach affektiven, evaluativen und sozialen Bedingungen phänomenologischer Forschung fragen und insofern zu deren Systematik beitragen sollte. An einer Ethik des phänomenologischen Denkens zu arbeiten, könnte darüber hinaus auch eine erneute ideengeschichtliche Re-Vision der verschiedenen phänomenologischen Philosophien und ihrer wechselseitigen Beziehungen in Gang setzen. Denn die Frage nach einem „Ausgleich“^65^ alternativer Phänomenologien bzw., generell, Philosophien kann sich retrospektiv, von verschiedenen historischen Gegenwarten der Selbstreflexion her, in neuem Licht stellen und zu neuen Einsichten führen. Das Thema des Ausgleichs ist eine intern-politische Angelegenheit jedes Denkens, das sich um Perspektivierung,^66^ Objektivität und Integration bemüht.

Ich schließe mit einer Vision: Eine gemäß diesem Aufgabenkatalog politisierte Phänomenologie wäre imstande, den Raum für (Gesellschafts-)Kritik und Selbstkritik beträchtlich zu erweitern. Das wäre ein wertvoller Beitrag zur Entwicklung unserer Denkkultur als einer individuellen und kollektiven Praxis.

## Footnotes

1 Vgl. Medina (2013) und Lee (2019).

2 Vgl. Husserl (1976b, 59): „Das Verständnis der Anfänge ist voll nur zu gewinnen von der gegebenen Wissenschaft in ihrer heutigen Gestalt aus, in der Rückschau auf die Entwicklung. Aber ohne ein Verständnis der Anfänge ist diese Entwicklung als *Sinnesentwicklung* stumm. Es bleibt uns nichts anderes übrig: wir müssen im ‚Zickzack ‘ vor- und zurückgehen; im Wechselspiel muss eins dem andern helfen. Relative Klärung auf der einen Seite bringt einige Erhellung auf der anderen, die nun ihrerseits auf die Gegenseite zurückstrahlt.“ Vgl. Günzel (2004) und Rinofner-Kreidl (2014, 42). In Husserl (2002, 391–396), wird das „Zickzack“ nicht als ein Verfahren gegenstandsgerichteter deskriptiver Analyse, sondern als Selbstklärung und -begründung der phänomenologischen Methode entwickelt.

3 Vgl. Gordon (1995), Krasnodebsky (2002), Derrida (2003), Moran (2011), Marder (2014) und Mignolo (2021).

4 Diese Kritik richtet sich nicht primär gegen eine phänomenologische Erkenntniskritik. Sie richtet sich generell gegen traditionelle Erkenntnistheorien, die nicht als soziale bzw. nicht-ideale Epistemologien ausgewiesen sind. Vgl. Harding (2003, v. a. 182–185), Kidd, Medina & Pohlhaus (2017), Ashton et al. (2020) und McKenna (2023). Demgegenüber verteidigt z. B. Carr (2022) eine ideale Epistemologie.

5 Vgl. Elster (1979), Hollis & Lukes (1982), Schnädelbach (1984), Hollis (1987), Davidson (2004), Robbins & Aydele (2009).

6 Vgl. Waldenfels (2008) und Canguilhem (2012).

7 Vgl. z. B. Crisp & Slote (1997), Statman (1997) und Weiss, Murphy & Salamon (2020).

8 Vgl. Thompson & Embree (2000), Ahmed 2006, Held (2012), Gurley & Pfeifer (2016), Jung & Embree (2016), Foti & Kontos (2017), Bedorf & Herrmann (2020a), DeRoo (2022) und Herrmann et al. (2024).

9 Vgl. Medina (2013) und Edenberg & Hannon (2021). Das Problem der „testimonial injustice“—der exkludierenden Praxis *und* Konzeptualisierung epistemischen Vertrauens—nimmt etwa in Miranda Frickers Konzeption der epistemischen Ungerechtigkeit breiten Raum ein. Vgl. Fricker (2007) und Guenther (2017).

10 Diese Frage greift Überlegungen auf, die Merleau-Ponty mit Blick auf Husserls transzendentale Phänomenologie in seiner *Phänomenologie der Wahrnehmung* formuliert. Das dabei leitende kritische Phänomenologie-Verständnis skizziert Merleau-Ponty bereits im Vorwort. Vgl. Merleau-Ponty (1966, 3–18, insbes. 6–12, 18).

11 Speziell mit der Horizontstruktur von Werterfahrungen befasse ich mich in Rinofner-Kreidl (2025a). Die oben genannten Begriffe verweisen auf einen Strukturzusammenhang, der in verschiedenen Arten von Erfahrung präsent ist. Im vorliegenden Kontext interessieren vor allem die drei erstgenannten Begriffe.

12 Zum im Idealfall gelingenden, womöglich aber in modalisierter oder aufgehobener Seinsgeltung terminierenden Bewährungsprozess als eines Strebens nach Selbstgegebenheit der Sache (a) in der Wahrnehmung, vgl. Husserl (1973b, 88–153 (§§ 27–43), v. a. 141f. (§ 40) u. 285–293 (§ 84)), Husserl (1966, 65–116 (§§ 16–25), auch zu *sekundärer Bewährung* (qua holistischer „Systembindung“ und Kontextualität): 98–100 (§ 22)); (b) in der Wertnehmung, vgl. Husserl (2020, 22–32, 40, 273–286, 397, 402–416, 536, 540–550); (c) mit Bezug auf Urteile, vgl. Husserl (1974, 126–131 (§ 44)), Husserl (1985, 339–352 (§§ 68–71)). Im Fall der Gemütsintentionalität erfordert Evidenzprüfung auch die „Erziehung des Geschmackssinnes“ und einen reflektierten Umgang mit Experten-Autorität, der verhindern soll, dass bloß „anempfundene Gefühle“—uneigentliche, nicht in eigenem Erleben begründete und „wohlfundierte“—mit einem wirklichen Gefallen (Wertschätzen) verwechselt werden. Letzteres ist ein objektiv richtiges, in der Sache selbst gründendes Gefühl. Vgl. Husserl (2020, p. 278, 283f.).

13 Wie im Folgenden zu verdeutlichen sein wird, ist damit nicht der epistemische Wert von Dissens bestritten: *was* die zu erforschende Sache ist, ist ja erst zu ermitteln. Im ersten Schritt ist jedoch anzuerkennen, dass es der Gegenstand selbst ist, der jede Erfahrung von ihm und jede gegenstandsbezogene Aushandlung bestimmter Auffassungen von ihm anleitet, *wenn Dissens nicht beliebig produziert und auf rationalem Weg prinzipiell unauflösbar sein soll.* Dass es bestimmte Formen von Dissens, sogenannte *deep disagreements*, gibt, für die eben das gilt, ist damit nicht in Abrede gestellt. Bestritten ist lediglich, dass dies die generelle Dissens-Situation unserer Erkenntnissuche darstellen könnte. Ebenso ist mit Obigem nicht in Abrede gestellt, dass mit Bezug auf gegebene—und wechselnde—theoretische oder praktische Interessen die Anforderungen daran, was als zureichende Erkenntnis der betreffenden Sache verstanden ist, variieren (können). Diese Variation erfolgt jedoch in einem gewissen Spektrum, das nicht beliebig ist, vielmehr in Art und Reichweite durch die Natur des Erkenntnisgegenstandes bestimmt ist.

14 Vgl. Husserl (2008, 195–199, 215–218, 637–672, 714–733), Husserl (1973a, 133–170) und Doyon & Breyer (2015).

15 Zum Umgang mit fallibler Evidenz vgl. Fn. 13. Auf der Meta-Ebene methodischer Reflexion schlägt sich das Bekenntnis zur Selbstbegrenzung bzw. zur fortschreitenden Klärung und insofern Überschreitbarkeit gegebener Evidenzen in einem Detail der Formulierung des *Prinzips aller Prinzipien* in § 24 im ersten Band der *Ideen* nieder: *„Am Prinzip aller Prinzipien: daß jede originär gebende Anschauung eine Rechtsquelle der Erkenntnis sei, daß alles,* was sich uns *in der ‚Intuition‘ originär,* (sozusagen in seiner leibhaften Wirklichkeit) *darbietet, einfach hinzunehmen sei, als was es sich gibt, aber auch nur in den Schranken, in denen es sich da gibt*, kann uns keine erdenkliche Theorie irre machen.“ (Husserl 1976a, 51, kursiv im Original) Dass Evidenz zwar das unhintergehbare Fundament („Rechtsquelle“) aller Erkenntnis ist, als solche aber zugleich eine kritische Prüfung ihrer Geltungsart und Reichweite verlangt, welche je nach Art der zu erkennenden Gegenständlichkeit, je nach Erkenntnisinteresse und Kontext variieren, ist in der Phrase angezeigt: „aber auch nur in den Schranken, in denen es sich da gibt.“

16 Vgl. Husserl (1985, 340f. (§ 68)).

17 Formal ist der Standort des Subjekts auch im phänomenologischen *Korrelationsapriori* impliziert, das eine nicht-zufällige Entsprechung zwischen bestimmten Typen der Erfahrung und bestimmten Arten von Gegenständen feststellt, sofern als Normierungsmaß die bestmögliche Erkennbarkeit des Gegenstandes zugrunde gelegt wird. Zu entdecken ist dieser Wesenszusammenhang nur dann, wenn im Sinne eines Gedankenexperimentes zugehörige Einstellungsänderungen durchlaufen werden. Siehe auch Fn. 32.

18 Zu den methodischen Rahmenbedingungen, welche die Differenz von transzendentaler (Inter-)Subjektivität und Subjektivismus sicherstellen, siehe Fn. 33.

19 Was künstlerische und ästhetische Wahrnehmung zu einem exemplarischen Fall nicht-normaler Wahrnehmung macht und in ein Näheverhältnis zu philosophischer Reflexion bringt, ist, dass sie den selbstbezüglichen Aspekt pointiert und die Transparenz der Wahrnehmung thematisiert, anstatt sie bloß zu „agieren“. Vgl. Husserls Brief an Hugo von Hoffmannsthal vom 12. Januar 1907, auf den mich dankenswerter Weise ein anonymer Reviewer hingewiesen hat. Vgl. Husserl (1994, 133–136).

20 Ich greife im Folgenden nur jene Aspekte heraus, die für die vorliegenden Zwecke relevant sind. Anderes (z. B. die lerntheoretischen Implikationen von Husserls Einstellungslehre) lasse ich beiseite.

21 Ein diesbezüglicher Konsens wie auch Dissens ist (moral-)epistemologisch nur dann relevant, wenn wir voraussetzen, dass geteilte gegenständliche Referenz möglich ist.

22 Das hier entworfene Bild des Erkenntnis- und Handlungssubjekts lässt Wesentliches offen. Es provoziert den Einwand der Idealisierung, da nur jene Fähigkeiten und Einsichten angesprochen werden, welche ausgewählten Strukturmerkmalen der menschlichen Erfahrung entsprechen. Die offenkundigen Lücken betreffen u. a. Fehlleistungen und Ambivalenzen im faktischen Gebrauch von Vernunftfähigkeiten. Diese unerörtert zu lassen, ist jedoch unvermeidlich, solange lediglich Formen der Erfahrbarkeit, Ermöglichungsbedingungen der Gegenstands- und Selbsterfahrung, im Fokus stehen. Daraus kann nicht geschlossen werden, dass die vielfältigen Verhinderungsbedingungen und Widerstände gegen rationale Überzeugungsbildung und -änderungen ignoriert würden bzw. die Tendenz vorherrschte, die Frage nach Erkenntnisprivilegien und -grenzen u. dgl. übergehen zu wollen.

23 Zur näheren Ausführung des Konzepts eines erweiterten Selbst vgl. Rinofner-Kreidl (2025a), v. a. Abschn. II.2.4.3: Axiologisch fundierte Selbsterkenntnis und die Rolle der Anderen. Dessen entscheidende Anforderung formuliert Dan Zahavi als „a balance between an undersocialized and an oversocialized view of the subject“, was die Verteidigung einer „nonrelational perspectival autonomy“ bei gleichzeitiger Anerkennung der sozialen Formung des Selbst erforderlich macht. Letztere ist jedoch nicht als vollständige soziale Konstitution des Subjekts zu verstehen. Vgl. Zahavi (2023, 10). Siehe unten Abschnitt 4 zur Notwendigkeit, epistemische und soziale Autorität zu unterscheiden.

24 Diese Einsicht spielt bereits in Adam Smiths *impartial spectator* eine zentrale Rolle. Vgl. Smith (1982). Zum Konnex mit Husserls Methodologie vgl. Fricke & Føllesdal (2012).

25 Mit Blick auf Charakterbildung wird in diesem Zusammenhang auch von Offenheit für Kritik, von „humility and learning“ gesprochen: „In order to fulfill yourself, you have to forget yourself. In order to find yourself, you have to lose yourself.“ (Brooks 2015, x). Auch wenn die verschiedenen Wissenskontexte und Textsorten sorgsam zu beachten sind (vgl. ebd., xiv-xv), ist es schwerlich bloß ein Zufall, dass die Forderung, sich in Selbsterneuerung und Selbsterweiterung mittels Selbstüberschreitung zu üben, nicht nur in lebenspraktischen, sondern auch in theoretischen Zusammenhängen eine zentrale Rolle spielt. In letzteren, z. B. im § 64 der *Cartesianischen Meditationen*, nimmt Selbstüberschreitung die Gestalt universaler Selbstbesinnung und intermonadischer Selbsterkenntnis an: „Das an sich erste Sein, das jeder weltlichen Objektivität vorangehende und sie tragende, ist die transzendentale Intersubjektivität, das in verschiedenen Formen sich vergemeinschaftende All der Monaden.“ (Husserl 1950, 182) Husserl schließt seine *Cartesianischen Meditationen* mit einer Feststellung, welche die phänomenologische Methodenlehre an antike Weisheitslehren annähert: „Das Delphische Wort γνωθι σεαυτόν hat eine neue Bedeutung gewonnen. Positive Wissenschaft ist Wissenschaft in der Weltverlorenheit. Man muß erst die Welt durch ἐποχή verlieren, um sie in universaler Selbstbesinnung wiederzugewinnen. *Noli foras ire*, sagt Augustin, *in te redi, in interiore homine habitat veritas*.“ (ibid., 183) Ein „Weltverlust“ ist das nur dann nicht, wenn die psychologische Innenschau durch philosophische Selbstbesinnung auf Basis phänomenologischer Reduktion ersetzt, die Phänomenologie als reine Korrelationsforschung betrieben und das Selbst als ein transzendental-intersubjektiv (oder „intermonadisch“) erweitertes verstanden wird.

26 Zur Wahrhaftigkeit dieses Strebens und zur Wahrheit als *transcendental good* und deren politischer Bedeutung vgl. Drummond (2003, v.a. 7ff.). In praktischen Kontexten kann „jemand Bestimmter werden“ auch als „für etwas Bestimmtes stehen“ erläutert werden. „To have integrity is to understand that one’s own judgment matters because it is only within individual person’s deliberative view points, including one’s own, that what is worth our doing can be decided. Thus, one’s own judgment serves a common interest of co-deliberators. Persons of integrity treat their own endorsements as ones that matter, or ought to matter, to fellow deliberators.“ (Calhoun 1995, 258) Vgl. Guenther (2017, 200).

27 Unter dem Titel „Erneuerung“ ist dies ein Leitthema in Husserls *Kaizo*-Artikeln. Vgl. Husserl (1989, 3–94), v. a. 20–43 (*Erneuerung als individualethisches Problem* (1924), 43–59 (*Erneuerung und Wissenschaft* (1922/23).

28 „Kritisch“ leitet sich vom griechischen Verb κρίνειν (*krínein)* her, das im Deutschen „unterscheiden“ oder „trennen“ bedeutet.

29 Zu den Spezifika einer so konzipierten phänomenologischen Theorie der Vernunft gehört gemäß Husserls *Analogie der Vernunft*, dass dieser „rational constraint“ auch für Manifestationen der axiologischen Vernunft, d. h. für die Vollzüge intentionalen Fühlens, gilt. Vgl. Rinofner-Kreidl (2015a) und (2022).

30 Z. B.: Migration und multikulturelle Gesellschaften; Verteilungsgerechtigkeit im Kontext der Klimaethik; Einschränkungen individueller Handlungsfreiheit zur Durchsetzung gesundheitspolitischer Maßnahmen; Hassrede (*Hate speech*) und andere öffentlichkeitswirksame affektive Phänomene, welche Defizite der Regulierung und Kultivierung von Gefühlen zu Tage bringen; die unzureichende vertikale Durchlässigkeit von Bildungssystemen; die Idee eines bedingungslosen Grundeinkommens angesichts technologischer Umbrüche am Arbeitsmarkt und entsprechend notwendiger Revisionen des Konzepts von Arbeit.

31 „Außersachlich“ bezieht sich auf die implizite Zurückdrängung und Abwertung des Bezugs auf die Sache und deren Eigencharakter infolge von i) und ii). Die Debatte beschränkt sich nicht auf politische und weltanschauliche Kontexte. Analoge Probleme stellen sich bezüglich psychologischer Aspekte der Bedeutungskonstitution und Überzeugungsbildung bzw. in diese gelegentlich hineinspielender psychischer Bedürfnislagen. Ersteres wird im Psychologismusstreit zu Beginn des 20. Jahrhunderts thematisiert, letzteres z. B. in rezenten Debatten über Selbsttäuschung und irrationale (irreguläre) Formen der Überzeugungsbildung. Vgl. Williams 1973, 136–151, Pears 1982, Davidson 2004, 169–212; Rinofner-Kreidl 2019.

32 Hier kann ich auf den anhängenden Klärungsbedarf nicht eingehen. Ich habe dies andernorts getan. Vgl. Rinofner-Kreidl (2000, 129–246, 686–785).

33 Zu den internen Politisierungseffekten einer professionellen, insbesondere institutionellen Formung von Erkenntnissubjekten vgl. auch May (1992) und (1996).

34 A fortiori ist der Anspruch auch nicht, über die Belange politischer Philosophie im Allgemeinen oder über Politiktheorie und Politikwissenschaft zu handeln.

35 In anderem Kontext habe ich diesbezüglich die AID (*Attitude, Intuition, Description*)-These formuliert, dass Einstellung, Anschauung und Beschreibung gemäß Husserls Methodologie in einem unauflöslichen Wechselverhältnis stehen, was einen naiven Intuitionismus effektiv unterbindet. Vgl. Rinofner-Kreidl (2014). In dieselbe Richtung zielt der oben skizzierte kritische Umgang mit Evidenz im Rahmen von Bewährungsprozessen.

36 Gemäß der hier vertretenen Unterscheidung von interner und externer Politisierung ist das phänomenologische Bekenntnis zu einem Standpunktpluralismus nicht mit einer Standpunkt-Epistemologie gleichzusetzen. Vgl. Anderson (2020) und Harding (2003).

37 Vgl. Husserl (1952, 268f.): „Die Entwicklung einer Persönlichkeit wird durch den Einfluß anderer bestimmt, durch den Einfluß fremder Gedanken, fremder aufsuggerierter Gefühle, fremder Befehle. Der Einfluß bestimmt die persönliche Entwicklung, ob die Person selbst später etwas davon weiß, sich daran erinnert, den Grad und die Art des Einflusses selbst zu bestimmen vermag oder nicht. […] Das Fremde, von mir ‚Übernommene ‘, mehr oder minder Äußerliche kann charakterisiert sein als vom fremden Subjekt ausgehend, zunächst als von ihm ausgehende und an mich sich wendende Tendenz, als *Zumutung*, der ich ev. passiv nachgebe, ev. widerwillig, doch bezwungen. Ev. aber eigne ich es mir selbsttätig an, und es wird zu meinem Eigentum. Es hat nun nicht mehr den bloßen Charakter einer Zumutung, der ich nachgebe, die mich von außen bestimmt; es ist eine Stellungnahme geworden, die von meinem Ich ausgeht, nicht ein bloßer Reiz, der darauf hingeht […]. Die *Autonomie der Vernunft*, die ‚Freiheit ‘ des personalen Subjekts besteht also darin, daß ich nicht passiv fremden Einflüssen nachgebe, sondern aus mir selbst mich entscheide. Und ferner darin, daß ich mich nicht von sonstigen Neigungen, Trieben ‚ziehen ‘ lasse, sondern frei tätig bin, und das in der Weise der Vernunft. Wir haben also zu unterscheiden zwischen der *menschlichen Person*, der apperzeptiven Einheit, die wir in der Selbstwahrnehmung und Wahrnehmung anderer erfassen, und der *Person als dem Subjekt der Vernunftakte*, deren Motivationen und Motivationskräfte uns im ursprünglichen eigenen Erleben sowie im nachverstehenden Erleben anderer zur Gegebenheit kommen. Dabei geht der Blick auf das spezifisch Geistige, das freie Aktleben.“

38 In anderer Hinsicht bedingt die Methode keineswegs eine Entindividualisierung. Sobald sich ein Subjekt zur fraglichen Methode und den ihr zugrunde liegenden Erkenntniszielen bekennt, wirkt dieses *commitment* individualisierend: Es ist ein Faktor, der das forschende Subjekt in einem bestimmten Ausmaß zu dem macht, was es ist—auch wenn das eine praktische Bestimmung ist, die sich im Umgang mit Theorien äußert, mithin im weiteren Sinn einer Ethik der Überzeugungen angehört. Der Unterschied zu jenen, welche *von bestimmten gesellschafts- und kulturpolitischen Standpunkten aus* immanenter Theoriearbeit Neutralismus vorwerfen, liegt darin, was als zulässige Quelle der Individualisierung der Erkenntnissubjekte anerkannt wird. Bei jenen, die den Neutralismus als (extern konstatierte) falsche Politik der Theoriearbeit bekämpfen, sind nur solche (z. B. gender-, ethnien- und herkunftsbezogene) Bestimmungsmerkmale von Subjekten zulässig, welche unabhängig von der betreffenden Theoriebildung gegeben sind.

39 Die oben unter dem Titel „interne Politisierung“ umrissene Methodengestalt phänomenologischer Forschung, die eine sukzessive Annäherung an das Gegebene in einem perspektivisch strukturierten Erfahrungsprozess ebenso inkludiert wie Fallibilität, Selbstdistanzierung und Standpunktpluralismus, eröffnet ein Verständnis von *phänomenologischer Kritik*, das sich mit jenem deckt, das in Loidolt (2022, insbes. 242–245) und Jansen (2022) entwickelt wird.

40 Vgl. Nagel (1986) und Wallace (2013).

41 Siehe Abschnitt 2.

42 Ein Beispiel aus eigener Forschung: In einer Studie zu Stolz und Hochmut, die sich u. a. mit der Plastizität und Verwandlungsfähigkeit dieser Gefühle befasste, arbeitete ich auch mit einem literarischen Beispiel: Henrik Ibsens *Hedda Gabler*. Dieses wurde zunächst rein immanent rekonstruiert und interpretiert—aus den sprachlichen Qualitäten des Textes, den Charakterzeichnungen, der Dialektik der Figurenkonstellation und ihrer affektiven Aufladung, dem Handlungsablauf und dessen dramatischer Zuspitzung. Dem gegenüber musste dann jedoch auch verdeutlicht werden, dass sich der Hochmut-Charakter Heddas anders darstellt, eine andere inhaltliche Pointierung, aber auch Tragik erhält, sobald eine feministisch-gesellschaftskritisch motivierte Lesart zugrundegelegt wird. Vgl. Rinofner-Kreidl (2025b).

43 Vgl. Nagel (1997, 17): „We cannot expect these matters to be settled permanently, since it is always possible that someone will come up with a new hypothesis explaining the perspectival character of some hitherto unassailable form of reasoning—or, on the contrary, that someone will come up with a credible way to extend reasoning to a new domain, like aesthetics.“ Siehe auch ibid., 21f.

44 *Nota bene*: Externe Politisierung wird hier nicht *tout court* mit Gefahren assoziiert und zurückgewiesen. Die Frage wird je nach Art der Externalisierung (radikal oder nicht) differenziert behandelt. In ihrer Zielrichtung deckt sich diese Differenzierung mit Miranda Frickers Zugang zu Fragen der epistemischen Ungerechtigkeit. Auch die Phänomenologie hat ein genuines Interesse daran, ein Mittelfeld auszumachen, auf dem Extrempositionen unterlaufen und die anhängenden Fehler vermieden werden—nämlich die Fehler „both of the ‚traditionalist ‘ (who regards the socio-political as irrelevant to epistemology) and of the ‚reductivist ‘ (who regards reason as just another form of social power).“ (Fricker 1998, 159) Vgl. ibid., 160.

45 Eine realpolitische Verwerfungslinie, die in der obigen Argumentation angelegt ist, besteht darin, dass sich radikale externe Politisierungen (z. B. in Gestalt politischer Aktion) keineswegs Vernunftansprüchen unterwerfen *müssen*. Dass dies angestrebt wird, erscheint sogar unwahrscheinlich, sofern die damit einhergehende Verpflichtung auf Begründbarkeit, einsichtige Argumentation und Selbstkritik als der realpolitischen Durchsetzungsmacht abträglich eingeschätzt wird. Ich danke Thomas Bedorf für einen diesbezüglichen Hinweis. Es liegt in der Natur der Vernunft, dass ihre Anerkennung nicht mittels äußerem Zwang, vielmehr letztlich nur als Vernünftig-sein-wollen der Akteure erfolgen kann. „Letztlich“ verweist darauf, dass typischerweise soziale Prozesse zwischengeschaltet sind, die die Entwicklung und Förderung der entsprechenden Fähigkeiten betreffen. Wir nennen diese Prozesse „Erziehung“, „Bildung“ oder „Aufklärung“. Ich kann hier auf dieses für die möglichen Zukünfte unserer Welt zentrale Thema nicht eingehen. Die Aufgabe besteht darin, nachkommenden Generationen nahezubringen, dass das, was sich als eine scheinbare Schwäche der Vernunft darstellt–nämlich dass sie nicht ein automatisch wirksamer Bauteil menschlicher Physiologie ist, sondern vielmehr der Kultivierung bedarf und auf individuelle Aneignung, gemeinschaftliche Ratifizierung und lebenslange Entwicklung angewiesen ist –, als eine Stärke der menschlichen Spezies zu sehen, in der ihr Potential an Kreativität und Überlebensfähigkeit liegt.

46 Auch unter tugendepistemologischen Gesichtspunkten des Fähigkeitserwerbs und des Lernens aus Erfahrung sind Denkmaterien relevant. Analoges gilt für epistemische Laster. Vgl. Cassam (2019). Materiale Bindungen bzw. Denkverpflichtungen, insbesondere wenn sie habitualisiert und biografisch „petrifiziert“ sind, können jedoch auch dazu führen, dass die Fähigkeit zum vernünftigen, selbständigen und kritischen Denken verlernt wird. Lernen und Verlernen vollziehen sich in Form komplexer Verhaltensmuster, die die Subjekte holistisch in Anspruch nehmen: als intellektuelle, aber auch als sinnliche, fühlende, wertende und handelnde Wesen. Hier eröffnet sich ein weites gesellschaftspolitisches Aufgabenfeld der Philosophie: Erziehung zu klarem, folgerichtigem und diskursfähigem Denken ist eine politische Angelegenheit ersten Ranges. Siehe Abschnitt 5.

47 Obiges legt nahe, dass öffentliche Stellungnahmen zu politischen Angelegenheiten, sofern sie den Ansprüchen kritischer Vernunft genügen sollen, auch medien- und formatsensibel erfolgen müssen. Vorsicht ist insbesondere geboten, wenn Medium und/oder Format bzw. das unmittelbare politische Interesse einer Äußerung oder eines Äußerungszusammenhanges eine differenzierte, multiperspektivische Beurteilung ausblenden oder unterdrücken. Dies gilt z. B. für Protestaktionen, offene Briefe u. dgl., die darauf abzielen, konkrete politische Anliegen und Sichtweisen zu stärken. Vgl. die Feuilleton-Debatten um Stellungnahmen prominenter politischer Philosophinnen zu den Vergeltungsschlägen des israelischen Militärs im Gaza-Streifen nach dem Terrorangriff der Hamas am 7. Oktober 2023.

48 In diesem Kontext von „normativer Kraft“ zu sprechen, setzt voraus, dass zwischen Normativität und Normalität nicht unterschieden bzw. erstere auf letztere zurückgeführt wird.

49 Vgl. De George (1985) und Hampton (1998); im phänomenologischen Rahmen, *pars pro toto*, von Hildebrand (2021).

50 Werden derartige Funktionen und Zuschreibungen nicht als Momentaufnahmen verstanden, geht es vielmehr um einen Habitus der betreffenden Subjekte, kompliziert sich die Lage. Gerade in Situationen, die eine gleichzeitige Ausübung sozialer und epistemischer (und eventuell auch moralischer) Autorität zulassen bzw. fordern, ist es schwierig, im Konfliktfall eindeutige Prioritäten auf Basis einer Rollendifferenzierung auszumachen.

51 Die Formen des Oktroyierens und Manipulierens sind vielfältig. Sie bewegen sich oft in Uneindeutigkeiten und verunklären auf diese Weise das Rollen- und Akteursbewusstsein derer, die aktiv und passiv involviert sind. Auch mit Blick auf die hier interessierende Problematik verdienen diese Varianzen, weit über sozialpsychologische Untersuchungen hinaus, die Aufmerksamkeit interdisziplinärer Analystinnen. Zum Untersuchungsfeld gehören z. B. erzwungene (nicht-freiwillige und unfreiwillige) Compliance; strategische Allianzen; Anreize für Komplizenschaft; absichtliche Desorientierung; Täuschung und Erzeugung von Nichtwissen; sozial induzierte Selbsttäuschungen. Einige Überlegungen dazu stelle ich in Rinofner-Kreidl (2015b) an.

52 Vgl. Nagel (1997, 13–35).

53 Vgl. Husserl (1975) und (2004), Nagel (1997, 14–16), Ratcliffe (2002) und Rinofner-Kreidl (2020).

54 Das Obige markiert die doppelte Lesart von „Kritik der Vernunft“ im Sinne des *genetivus objectivus* (Vernunft als Gegenstand der Kritik) und *genetivus subjectivus* (Vernunft als Agens der Kritik). Nagels Problemformulierung weist Vernunftausübung als eine Leistung aus, die wesentlich in dieser Doppelung vorkommt.

55 Wäre das, was behauptet wird—die vollständige externe Determination des Denkens—, wahr, dann wären diese Wahrheit und Realität *für uns* notwendig unerkennbar. Der Idee einer epistemisch irreduziblen Erste-Person-Perspektive des Denkens („für uns“) käme keinerlei Bedeutung mehr zu; sie hätte keinen Anhalt in der Erfahrung und könnte auch nicht in einer Erfahrungsreflexion thematisiert werden, die voraussetzungsgemäß anderes wäre als Manifestation externer Determinanten.

56 In Husserls pathosgetragener Wissenschaftssprache: „[I]n *unserem* Philosophieren [sind wir] *Funktionäre der Menschheit*. Die ganz persönliche Verantwortung für unser eigenes wahrhaftes Sein als Philosophen in unserer innerpersönlichen Berufenheit trägt zugleich in sich die Verantwortung für das wahre Sein der Menschheit […].“ (Husserl 1976a, 1976b, 15). Vgl. Husserl (1989).

57 Vgl. Weiss, Murphy & Salamon (2020), insbes. die Einleitung der Hgg., ibid, xiii-xiv, und die Beiträge von Davis (3–9) und Guenther (11–16). Was aus Sicht der kritischen Phänomenologinnen dieser Dichotomisierung zuarbeitet, ist insbesondere die strikte Unterscheidung zwischen Husserls Phänomenologie und der „existential phenomenology“ Heideggers, Merleau-Pontys, Sartres und Beauvoirs. Eine differenziertere Sicht bietet jüngst der Sammelband *The Existential Husserl* (Cavallaro & Heffernan 2022). Auch in der gegenwärtigen Debatte um die kritische Phänomenologie erfolgt die Abgrenzung häufig ambivalent und nicht eindeutig zugunsten einer Diskontinuitätsthese: „[C]ritical phenomenology […] reflects on the structural conditions of its own emergence, and in this it is following an imperative that is both critical in its reflexivity and phenomenological in its taking-up of the imperative to describe what it sees in order to see it anew. In this, what is critical about critical phenomenology turns out to have been there all along.“ (Salamon 2018, 12).

58 In differenzierter Weise wurde die Kontinuitätsthese kürzlich von Lanei Rodemeyer entwickelt, deren Befund ich in allen wesentlichen Punkten zustimme. Vgl. Rodemeyer 2022.

59 Vgl. Guenther (2020, 15): „Critical Phenomenology goes beyond classical phenomenology by reflecting on the quasi-transcendental social structures that make our experience of the world possible and meaningful, and also by engaging in a material practice of ‘restructuring the world’ in order to generate new and liberatory possibilities for meaningful experience and existence. In this sense, critical phenomenology is both a way of doing philosophy and a way of approaching political activism. […] As a political practice, critical phenomenology is a struggle for liberation from the structures that privilege, naturalize, and normalize certain experiences of the world while marginalizing, pathologizing, and discrediting others. These structures exist on many levels: social, political, economic, psychological, epistemological, and even ontological.”  Vgl. Guenther (2017), 203.

60 Dasselbe gilt für die Feststellung: „What existential phenomenology [à la Hannah Arendt, SRK] lacks is the ability to reflect on the political foundation of its own concepts.“ (Bedorf & Herrmann 2020b, 7) Vgl. ibid.,11.

61 Vgl. Harding (2003, 168f.). Offenheit nach außen ist notwendig, um Denkroutinen und eingespielte Sichtweisen, womöglich auch einen zu selbstsicheren, selbstgefälligen Methodengebrauch aufzubrechen. Vgl. dazu, in anderem Kontext, aber hilfreich, was Eric Schwitzgebel über einen „noxiously comfortable view of ourselves“ ausführt: „Once we have our judgments right, we have our beliefs right, and thus we have right that aspect of our minds about which the philosophical community cares. It is thus easier than it should be to regard ourselves as free of racist, sexist, elitist, and other objectionable attitudes.“ (Schwitzgebel 2010, 547).

62 Gegen eine Verwissenschaftlichung der Politik wird u. a. vorgebracht, dass sie die normativen Ansprüche bzw. Wertverpflichtungen der Politik in den Hintergrund rückt bzw. vernachlässigt. Vgl. Bogner 2021. Umgekehrt steht auch die Politisierung der Wissenschaft im Fokus der Kritik, sofern sie als De-Professionalisierung im Sinne der Externalisierung von Kontrollmechanismen verstanden wird. Vgl. Weingart 1983. Eine vergleichende Betrachtung der Politisierung der Philosophie und der Politisierung von Wissenschaft, welche gesondert nach Wissenschaftstypen und disziplinenspezifischen Rahmenbedingungen durchzuführen wäre, konnte hier nicht angestrebt werden. Die vorliegende Argumentation legt jedoch nahe, dass die Sachlagen in relevanten Hinsichten verschieden sind. Letztlich dürfte dies darin begründet sein, dass die Fragen der Autorität—der Autonomie der Wissensproduktion und -anwendung—und des Vertrauens in die Selbstregulierung im Fall der Philosophie direkt die Vernunft und das unter dem Anspruch der Vernunft stehende Subjekt betreffen—mithin das, was in allen Wissenschaften und konkreten Erkenntnisverfahren und -praxen immer schon vorausgesetzt ist. Mitte der 30er Jahre des 20. Jahrhunderts nennt Husserl als letzten Grund für die existenzielle Krise der Wissenschaften (bei fortlaufend ungebrochener explanatorischer Kraft und Produktivität), dass der Glaube an die Vernunft verlorenzugehen drohe. Vgl. Husserl (1976a, 1976b, 9–17).

63 Vgl. Mignolo & Walsh (2018), Lee (2019), Heinämaa & Jardine (2021) und Mignolo (2021).

64 Das oben Genannte skizziert den dichten theoretischen Kontext, mit Bezug auf den von einer „politics of conceptualisation“ gesprochen werden kann. Vgl. die auf ein anderes Gegenstandsgebiet bezogenen, aber methodologisch kompatiblen und erhellenden Ausführungen dazu in Lindahl (2018, 2–6, 5), speziell zur Unausweichlichkeit einer Dialektik von Inklusion und Exklusion.

65 Vgl. Scheler (2015, 33–35, 77, 120f.). Ein zentraler Aspekt von Ausgleich ist *Depolarisierung*. Vgl. Cassam (2021).

66 Unter „Perspektivierung“ verstehe ich hier das Sichtbarmachen bzw. Bewusstmachen der Perspektivität des eigenen Denkens aufgrund der imaginären Übernahme fremder Perspektiven. Siehe oben Abschnitt 2.
